# Small Molecule Calcium Channel Activator Potentiates Adjuvant Activity

**DOI:** 10.1021/acschembio.1c00883

**Published:** 2022-01-05

**Authors:** Tetsuya Saito, Nikunj M. Shukla, Fumi Sato-Kaneko, Yukiya Sako, Tadashi Hosoya, Shiyin Yao, Fitzgerald S. Lao, Karen Messer, Minya Pu, Michael Chan, Paul J. Chu, Howard B. Cottam, Tomoko Hayashi, Dennis A. Carson, Maripat Corr

**Affiliations:** † Moores Cancer Center, University of California San Diego, La Jolla, California 92093-0809, United States; ‡ Department of Rheumatology, Graduate School of Medical and Dental Sciences, Tokyo Medical and Dental University (TMDU), Tokyo 113-8519, Japan; § Herbert Wertheim School of Public Health and Longevity, University of California San Diego, La Jolla, California 92093-0901, United States; ∥ Department of Medicine, University of California San Diego, La Jolla, California 92093-0656, United States

## Abstract

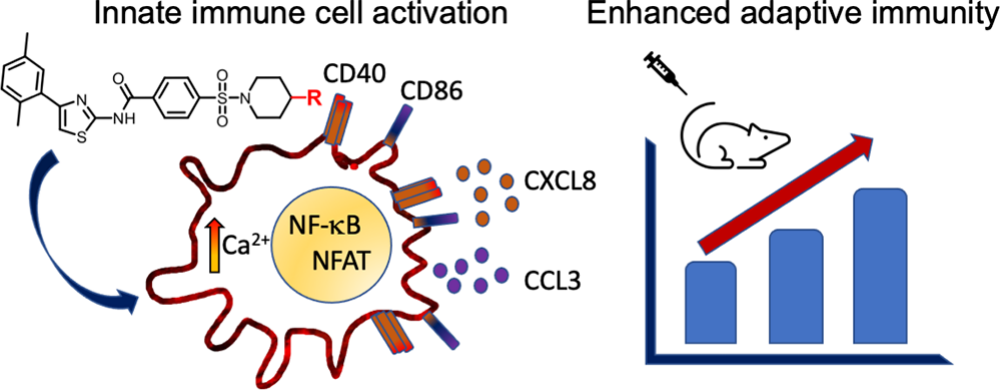

There remains an unmet need for reliable fully synthetic adjuvants that increase lasting protective immune responses from vaccines. We previously reported a high-throughput screening for small molecules that extended nuclear factor kappa-light-chain enhancer of activated B cells (NF-κB) activation after a Toll-like receptor 4 (TLR4) ligand, lipopolysaccharide (LPS), stimulation using a human myeloid reporter cell line. We identified compounds with a conserved aminothiazole scaffold including **2D216** [*N*-(4-(2,5-dimethylphenyl)thiazol-2-yl)-4-(piperidin-1-ylsulfonyl)benzamide], which increased murine antigen-specific antibody responses when used as a co-adjuvant with LPS. Here, we examined the mechanism of action in human cells. Although **2D216** activated the major mitogen-activated protein kinases, it did not interact with common kinases and phosphatases and did not stimulate many of the pattern recognition receptors (PRRs). Instead, the mechanism of action was linked to intracellular Ca^2+^ elevation via Ca^2+^ channel(s) at the plasma membrane and nuclear translocation of the nuclear factor of activated T-cells (NFAT) as supported by RNA-seq data, analysis by reporter cells, Ca^2+^ flux assays, and immunoblots. Interestingly, **2D216** had minimal, if any, activity on Jurkat T cells but induced cytokine production and surface expression of costimulatory molecules on cells with antigen-presenting functions. A small series of analogs of **2D216** were tested for the ability to enhance a TLR4 ligand-stimulated autologous mixed lymphocyte reaction (MLR). In the MLR, **2E151**, *N*-(4-(2,5-dimethylphenyl)thiazol-2-yl)-4-((4-propylpiperidin-1-yl)sulfonyl)benzamide, was more potent than **2D216**. These results indicate that a small molecule that is not a direct PRR agonist can act as a co-adjuvant to an approved adjuvant to enhance human immune responses via a complementary mechanism of action.

## Introduction

Vaccines are a key component of public health and are quintessential for protecting vulnerable populations from lethal contagious pathogens. As a critical component of vaccines, adjuvants enhance the magnitude and duration of protective immune responses without compromising safety. However, the development of new adjuvant systems has lagged this pressing need. Until the late 1990s, alum (aluminum salts) was the most widely used adjuvant.^1^ A squalene oil-in-water emulsion adjuvant, MF59, was then included in Fluad, a trivalent inactivated influenza vaccine approved for older adults.^2^ Similarly, AS03, another squalene oil-in-water emulsion that contains α-tocopherol (vitamin E), was used in influenza vaccines Pandemrix and Arepanrix in Europe and Canada, respectively.^3^

More recent investigations have focused on ligands for pattern recognition receptors (PRRs) including nucleotide-binding oligomerization domain-like receptors (NLRs), Toll-like receptors (TLRs), and retinoic acid-inducible gene-I-like receptors (RLRs) to use as innate immune adjuvants.^4−13^ Although most adjuvants are based on natural products, fully synthetic molecules may allow less rigorous storage conditions and be targeted to specific pathways as discussed above. Synthetic agonists for the TLRs have been identified as promising candidates including TLR 2 agonists,^14^ double-stranded RNA {polyinosinic:polycytidylic acid [poly(I:C)] stabilized with poly-l-lysine [poly(ICLC)]}, an agonist for TLR3 and melanoma differentiation-associated protein 5 (MDA5),^15^ glucopyranosyl lipid A (GLA),^16^ a TLR4 agonist, MEDI9197 and 3M-052, TLR7/8 agonists,^17,18^ and cytosine phosphoguanosine (CpG) 1018, a TLR9 agonist.^19^ Some of these agents are being developed as adjuvants for tumor vaccines,^17^ while the use of others has been focused largely on vaccines to prevent infections.^20,21^ The TLR9 agonist CpG 1018 has been successfully developed and FDA approved as the adjuvant for the hepatitis B vaccine, Heplisav-B.^19^

Synthetic agonists for other innate immune-activating molecules like the stimulator of interferon genes (STING) are also being developed as tumor vaccines or intratumoral immune activators including ADU-V19 (RR–S2 cGAMP)^22^ and ADU-S100 (ML RRS2 CDA or MIW815).^23^ ADU-S100 is undergoing phase I or phase II clinical trials (NCT03937141, NCT03172936, and NCT02675439). Lipid-A-derived compounds, including chitosan, saponins, and glucans, continue to be used as adjuvants;^24^ however, synthetic carbohydrates like monophosphoryl 3-deacyl lipid A (3D-PHAD) and 3-deacyl monophosphoryl hexa-acyl lipid A (3D-(6-acyl) PHAD) have been developed and used in HPV and HBV vaccines.^25^

In addition to targeted approaches for identifying innate immune ligands as adjuvants, there has also been a focus on employing combinations of approved adjuvants to enhance the potency of vaccines. Examples of co-adjuvant systems include Adjuvant System 01 (AS01) that contains monophosphoryl lipid A (MPLA) and an isolated and purified saponin fraction (QS-21) in the shingles vaccine, Shingrix,^26^ and AS04 that contains MPLA and alum and is approved in a hepatitis B vaccine, Fendrix, and human papillomavirus vaccine, Cervarix.^27−30^ Newer synthetic small molecule immune potentiators are being examined in combinations and with established adjuvants. They could be used as single adjuvants or in combination with other approved agents as co-adjuvants.^18,31^

Despite recent advances, our understanding of the mechanisms of action of the currently available adjuvants remains incomplete. Hence, in lieu of a targeted approach, we previously utilized a phenotypic approach with a broad-cell-based assay to capture previously unappreciated pathways.^32^ As the nuclear factor kappa-light-chain-enhancer of activated B cells (NF-κB) is a major downstream transcription factor of multiple PRR pathways, we identified that a small molecule prolonging a TLR4-initiated NF-κB signal could be a suitable co-adjuvant to enhance immune responses. We have reported a high-throughput screening (HTS) of a library of approximately 166,300 compounds in conjunction with lipopolysaccharide (LPS) using a human monocytic cell line, THP-1, equipped with a NF-κB reporter construct.^32^ To improve the reliability of identifying hit compounds, we employed a statistical clustering method to identify compounds with conserved scaffolds and noted that eight aminothiazoles in the original library increased the NF-κB activation by LPS.^33^ We selected one of these compounds (**2D216**) and found that it functioned as a co-adjuvant in mice and increased viral neutralizing titer sufficiently to protect mice from a lethal influenza challenge.^34^ Here, we aimed to examine the mechanisms of action in human cells and evaluated a small set of analogs to enhance activation of human immune cells.

## Results and Discussion

### **2D216** Acts as a Co-Adjuvant when Used with a TLR4 Ligand

**Figure 1:**
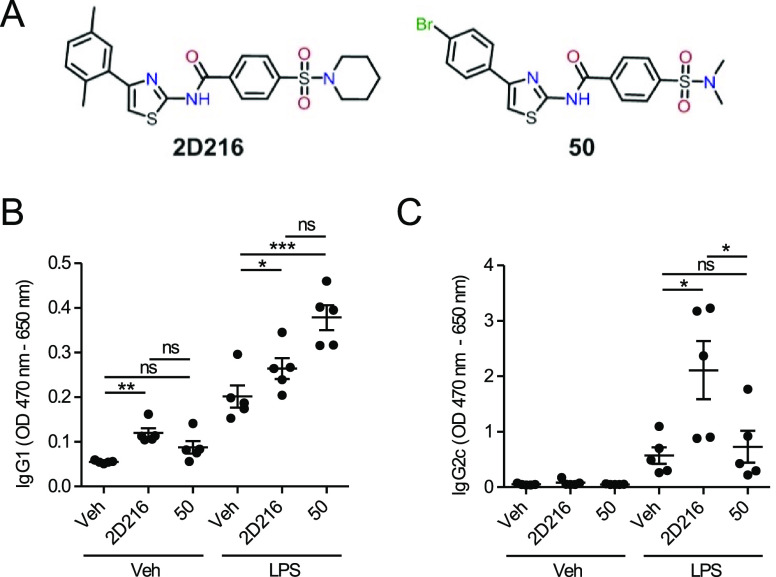
Aminothiazole compounds as TLR4 ligand co-adjuvants. (A) Chemical structures of two HTS-identified hit compounds with an aminothiazole scaffold, **2D216** and **50**. (B, C) Co-adjuvanticity of compounds **2D216** and **50** with LPS. Mice (*n* = 5) were immunized intramuscularly with an antigen (OVA, 20 μg/mouse), LPS (3 μg/mouse), and compound **2D216** or **50** (100 nmol/mouse) on days 0 and 7. The immunized mice were bled on day 17 and OVA-specific IgG1 (B) and IgG2c (C) antibody binding was measured using ELISA. Data (OD 470–650 nm) represent mean ± SEM. **p < 0.01, ***p < 0.001, ns (not significant) by one-way ANOVA with Tukey’s *post hoc* test.

To identify small molecules that would enhance the potency of approved TLR adjuvants, we previously conducted a HTS using LPS as a primary stimulus and tested a large library of compounds for ones that protracted the NF-κB signal in reporter cells.^32^ We examined the same compounds in two screens for NF-κB activation after 5 and 12 h of incubation and found an active cluster of compounds with a conserved aminothiazole scaffold.^35^ As proof of concept, compound **50** (*N*-(4-(4-bromophenyl)thiazol-2-yl)-4-(*N*,*N*-dimethylsulfamoyl)benzamide) and compound **2D216** (*N*-(4-(2,5-dimethylphenyl)thiazol-2-yl)-4-(piperidin-1-ylsulfonyl)benzamide (Figure 1A), which generated the highest levels of NF-κB reporter signals at 5 and 12 h respectively, were examined as potential co-adjuvants in mice with a prototypic TLR4 agonist, LPS, and ovalbumin (OVA) as a test antigen. Injection with compound **2D216** as a co-adjuvant resulted in greater levels of both antigen-specific IgG1 and IgG2c antibodies compared to LPS as a single adjuvant, whereas injection with compound **50** increased IgG1 but not IgG2c levels (Figure 1B,C). The most widely used FDA-approved adjuvant, alum, preferentially induces T helper (Th)-2-associated antibody responses (IgG1 in mice),^1,36^ but **2D216** with LPS also increased the IgG2c response (Th1 associated), indicating that LPS with **2D216** induced a more balanced immune response. We recently demonstrated that mice immunized with an inactivated influenza A virus adjuvanted with **2D216** in combination with another TLR4 ligand, MPLA, were protected from a lethal influenza challenge and had an increased antiviral neutralization titers.^34^ These results indicated that a **2D216** plus MPLA adjuvanted vaccine induced an effective antibody response. The induction of antigen-specific IgG2c antibodies with **2D216**, but not with LPS alone, suggested that the mechanism of action of **2D216** differed from the canonical TLR4 signaling pathways.

### **2D216** Increases LPS-Induced Activation of THP-1 Cells and Primary Human Immune Cells

**Figure 2:**
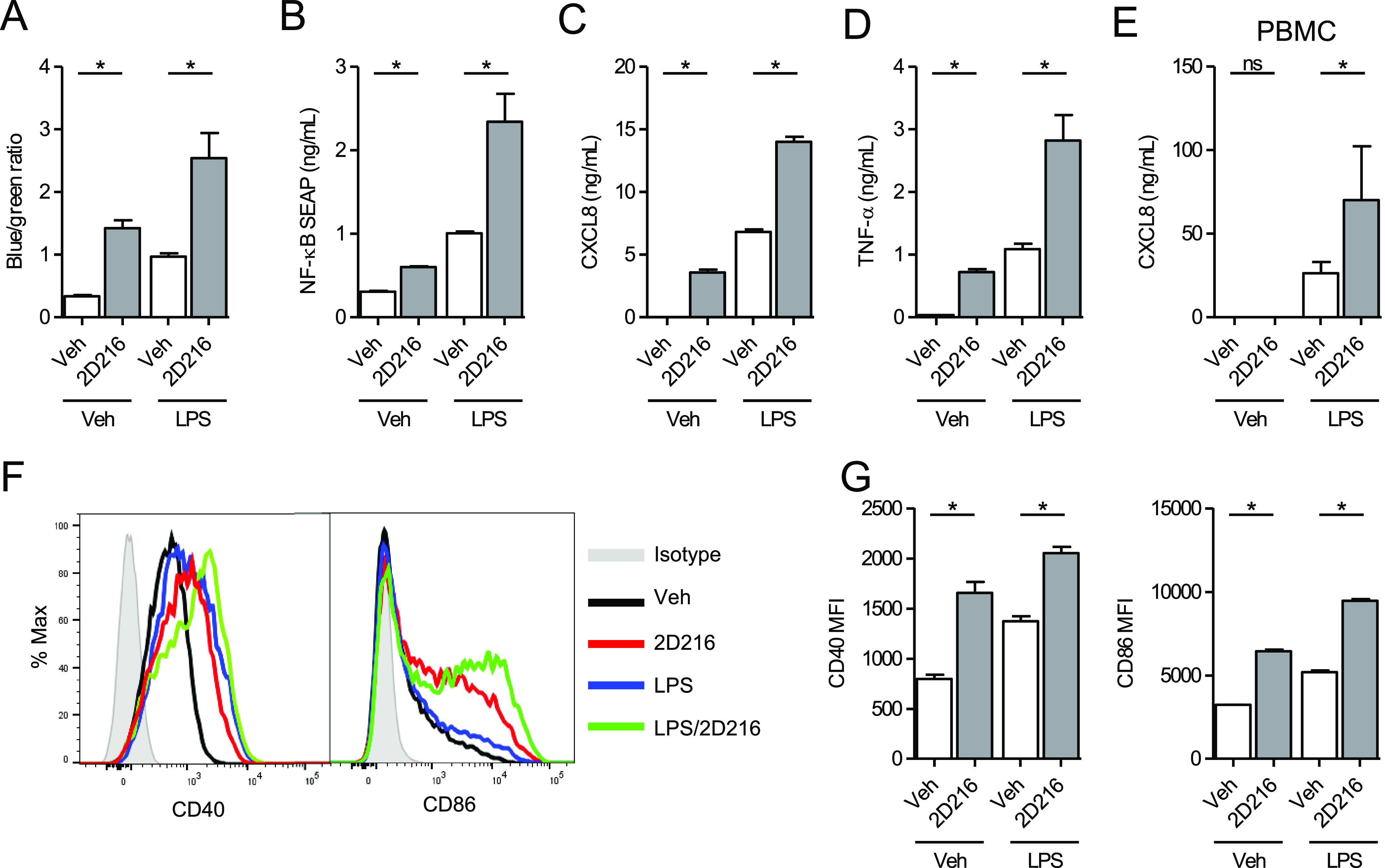
Characterization of immune activation by compound **2D216**. (A) CellSensor NF-κB-bla THP-1 reporter cells (0.5 × 10^6^ cells/mL) and (B) THP1-Blue NF-κB reporter cells (0.5 × 10^6^ cells/mL) were incubated for 16 h with vehicle (Veh), **2D216** (5 μM), LPS (10 ng/mL), or **2D216** (5 μM) plus LPS (10 ng/mL), and NF-κB activation was detected by FRET or SEAP in the culture supernatants, respectively. (C–E) CXCL8 and TNF-α secretion induced by **2D216**. (C, D) THP-1 cells (0.5 × 10^6^ cells/mL) or (E) human PBMC (0.5 × 10^6^ cells/mL) was incubated with Veh, **2D216** (5 μM), LPS (10 ng/mL), or **2D216** (5 μM) plus LPS (10 ng/mL) for 20 h, and the levels of CXCL8 and TNF-α in the culture supernatants were measured by ELISA. Data are presented as mean ± SD of triplicates and are representative of two independent experiments showing similar results. *p < 0.05 by Mann–Whitney *U* test. (F, G) Co-stimulatory molecule expression by **2D216**. THP-1 cells were incubated with Veh, **2D216** (5 μM), LPS (10 ng/mL), or **2D216** (5 μM) plus LPS (10 ng/mL) for 24 h and subjected to flow cytometry analysis with anti-CD40 and anti-CD86 antibodies. (F) Representative histograms. (G) Mean fluorescence intensity (MFI) for CD40 and CD86 relative to Veh is shown. Data presented are mean ± SD of triplicates and are representative of two independent experiments showing similar results. *p < 0.05 by the Mann–Whitney *U* test.

As compound **2D216** augmented both Th1- and Th2-associated antibody responses, it was selected as a lead molecule for this chemotype, and its function was further characterized in human cells. The ability of **2D216** to reliably induce NF-κB-mediated reporter activity alone or in combination with LPS was confirmed with two THP-1 NF-κB reporter cell lines. The assay using Förster or fluorescence resonance energy transfer (FRET) assessed the NF-κB signal at a single time point (here16 h), and the secreted alkaline phosphatase (SEAP) reporter assay measured the accumulation of SEAP in the supernatant over the 16 h time period (Figure 2A,B), suggesting that there was a sustained level of NF-κB activation beyond the usual time of decay of an LPS-induced signal.

The ability of **2D216** to stimulate the innate immune system in an adjuvant setting was tested by measuring the induction of proinflammatory cytokines CXCL8 (IL-8) and TNF-α production by THP-1 cells (Figure 2C,D). **2D216** was more effective in inducing these cytokines than CCL5 and CXCL10 that are associated with type I interferon induction (Figure S1). **2D216** also significantly enhanced the LPS-induced secretion of CXCL8 in primary human peripheral mononuclear cells (PBMC; Figure 2E). The activation of antigen-presenting cells (APC) in vivo is also marked by an increase in surface proteins including CD40 and CD86 that interact with costimulatory molecules on cognate T cells. **2D216** increased both CD40 and CD86 expression on the surface of THP-1 cells (Figure 2F,G). An increase in CD40 and CD86 is also associated with a maturational transition of THP-1 cells into a macrophage phenotype and a higher level of expression that occurs in the matured fraction.^37^ These data suggested that **2D216** could stimulate human innate immune cells with markers associated with priming adaptive immune responses.

### **2D216** Induces a Gene Signature Indicating Activation of MAPK and Ca^2+^/NFAT Pathways

**Figure 3:**
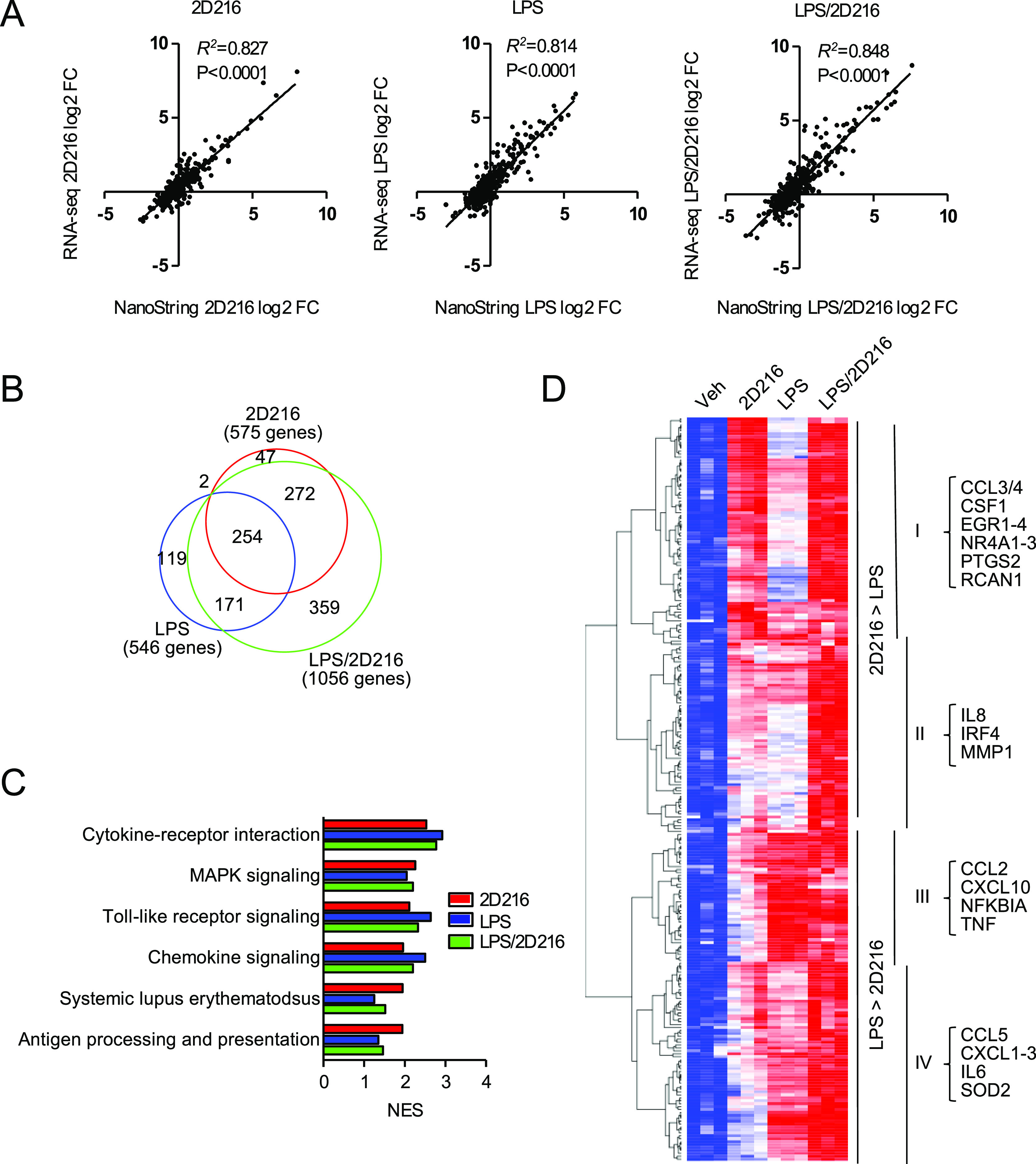
RNA-seq analysis of genes and pathways activated by **2D216**. (A) Correlations of a log2 fold change between RNA-seq and NanoString data. Dots represent the average log2 transformed values of fold changes for **2D216** vs Veh, LPS vs Veh, and LPS/**2D216** vs Veh for 5 h in RNA-seq (triplicate) and for 4 h in NanoString (duplicate). Pearson correlations and *p*-values are shown. NES: normalized enrichment scores. (B) Venn diagram of upregulated genes (log2 FC > 1 and FDR < 0.05) by stimulation with **2D216** (5 μM) and/or LPS (10 ng/mL) for 5 h in THP-1 cells. (C) KEGG pathway enrichment analysis of upregulated genes by **2D216** and/or LPS. Top six pathways upregulated by **2D216** (normalized enrichment score (NES) > 2 and FDR < 0.05) are listed. (D) Hierarchical clustering analysis of 254 genes commonly upregulated by **2D216**, LPS, or in combination. The representative genes involved in immune responses in each category are listed.

As an initial assessment for the mechanism of action of **2D216**, we examined the global gene expression induced by **2D216** in the presence and absence of LPS using RNA-seq analysis.^38^ The RNA-seq data were validated using a human nCounter Immunology Panel (NanoString Technologies) as an alternative assay, which showed strongly positive correlations (Figure 3A). Five-hour treatment of THP-1 cells with **2D216** upregulated 575 and downregulated 175 genes was compared to vehicle control, log2 fold change (FC) > 1 and false discovery rate (FDR) < 0.05. The individual numbers of genes upregulated by **2D216**, LPS, and the combination of LPS and **2D216** (LPS/**2D216**) are summarized in a Venn diagram (Figure 3B). Gene-enrichment and functional annotation analysis of upregulated genes by the combination of **2D216** and LPS showed that the genes upregulated by **2D216**/LPS were enriched for pathways related to cell surface, adhesion, and transcription molecules (Figure S2). In the Kyoto Encyclopedia of Genes and Genomes (KEGG) pathway database, gene set enrichment analysis of the upregulated genes by **2D216**, LPS, and LPS/**2D216** indicated that the top six enriched pathways upregulated by **2D216** alone were cytokine receptor interaction, mitogen-activated protein kinase (MAPK) signaling, TLR signaling, chemokine signaling, systemic lupus erythematosus, and antigen processing and presentation, which were also upregulated by LPS and LPS/**2D216** (Figure 3C). These pathways were most highly enriched in 254 gene transcripts that were commonly upregulated by **2D216**, LPS, and **2D216**/LPS (Figure 3B). When hierarchical clustering analysis was performed with these 254 transcripts, the expressed genes were divided into two clusters dominantly upregulated by **2D216** (Clusters I and II) and two clusters by LPS (Clusters III and IV) (Figure 3D). The genes highly upregulated by **2D216** in Cluster I, including C-C motif chemokine ligand (CCL) 3, are known to be regulated by the transcription factor nuclear factor of activated T-cells (NFAT).^39^

This expression signature correlated with the signaling pathway analysis performed with a panel of CellSensor cell lines where **2D216** activated NFAT (EC_50_ 540 nM) in a human B cell line (NFAT-bla RA1, Table S1). Hence, the RNA-seq analysis suggested that **2D216** may activate MAPK and NFAT pathways in addition to NF-κB. We next examined how these pathways are involved in the mode of action of **2D216**.

### **2D216** Activates the MAPK Pathway in THP-1 Cells

**Figure 4:**
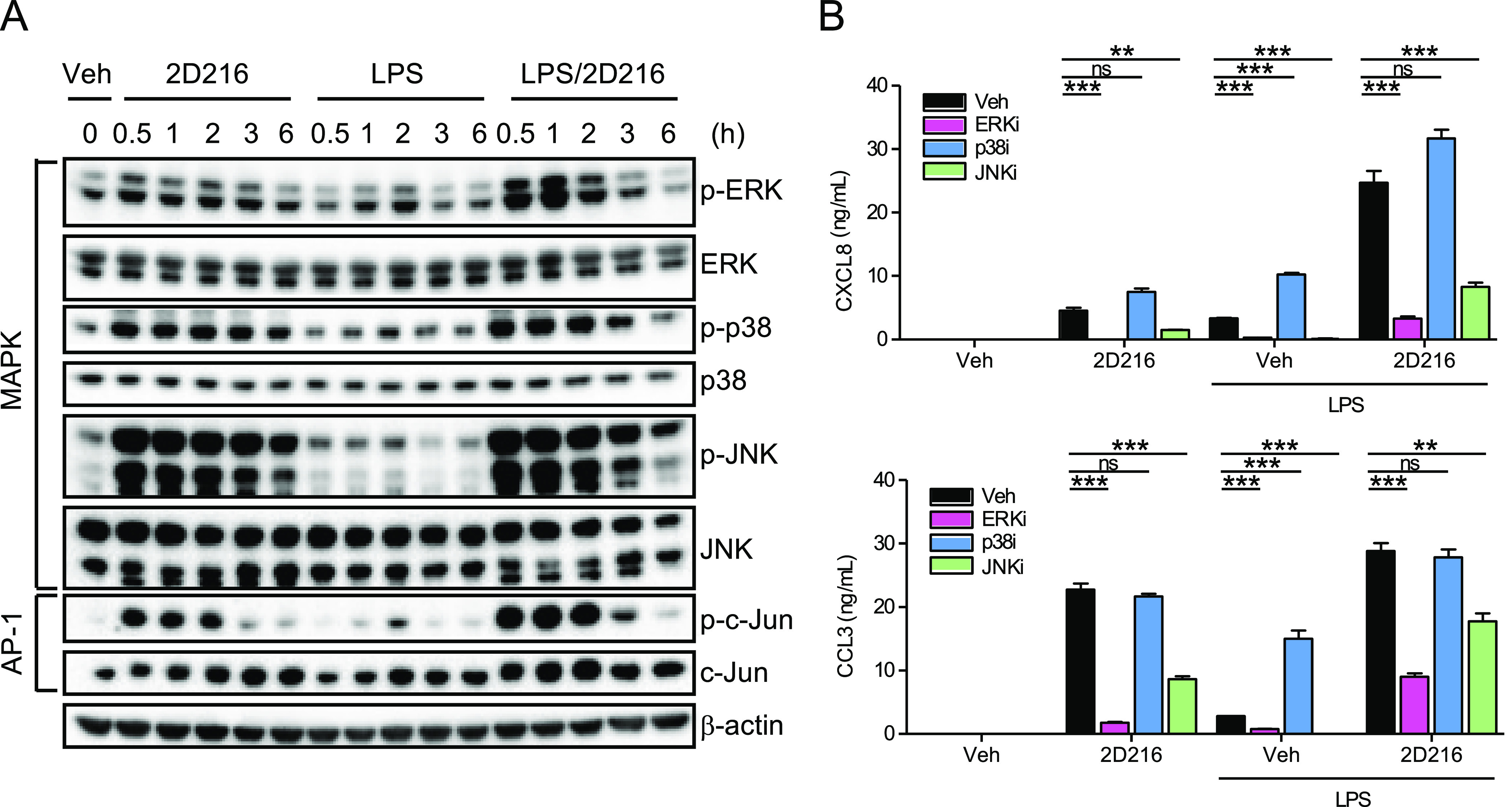
MAPK activation by **2D216**. (A) Immunoblot for activation of MAPK/AP-1 pathways. THP-1 cells were treated with **2D216** (5 μM), LPS (10 ng/mL), or **2D216** (5 μM) plus LPS (10 ng/mL) up to 6 h, and phosphorylations of MAPK (ERK, p38, and JNK) and c-Jun, a component of the AP-1 transcription factor, were detected by immunoblot with phospho-specific antibodies against each protein. (B) Cytokine secretion with MAPK inhibitors. THP-1 cells (0.5 × 10^6^ cells/mL) were pre-treated for 1 h with MAPK inhibitors, ERKi (SCH772984, 2 μM), p38i (SB203580, 10 μM), or JNKi (SP600125, 20 μM) and treated with **2D216** (5 μM) and/or LPS (10 ng/mL) overnight. CXCL8 and CCL3 in supernatants were determined by ELISA. Data represent mean ± SD of triplicates of two independent experiments showing similar results. *p < 0.05, **p < 0.01, ***p < 0.001, ns (not significant) by one-way ANOVA with Tukey’s *post hoc* test.

To validate that MAPKs are activated by **2D216**, we assessed the phosphorylation of three MAPKs [extracellular signal-regulated kinase (ERK), c-Jun N-terminal kinases (JNK), and p38 mitogen-activated protein kinase (p38)] and c-Jun, a component of activator protein-1 (AP-1), by immunoblot (Figure 4A). **2D216** stimulated phosphorylation of these MAPKs (ERK, p38, and JNK) as a single agent within 30 min, which lasted approximately 3 h, while the phosphorylation of MAPK by LPS peaked at 1 h. The additive effects of **2D216** and LPS were clearly observed in the phosphorylation of ERK and JNK. Similarly, **2D216** alone stimulated the phosphorylation of c-Jun within 30 min, and the combination with LPS further enhanced the phosphorylation (Figure 4A). ERK and JNK inhibitors (SCH772984 and SP600125, respectively) significantly suppressed CXCL8 and CCL3, which are regulated by NF-κB and NFAT, respectively,^39^ release by THP-1 cells stimulated with **2D216** alone or in combination with LPS. In contrast, the p38 inhibitor (SB203580) did not have any significant effects on CXCL8 or CCL3 production (Figure 4B). These data indicated that the ERK and JNK pathways are involved in **2D216**-induced CXCL8 and CCL3 production but p38 may be dispensable.

MAPK phosphorylation is regulated by diverse innate immune stimulators, including PRR activation. In the original HTS we utilized LPS as the primary stimulus and subsequently demonstrated that **2D216** could induce similar functions as a single agent (Figure 2A–D,F,G). **2D216** not only enhanced CXCL8 release from THP-1 cells stimulated with a TLR4 ligand (LPS) but also when combined with other PRR ligands including Pam3CSK4 (TLR2), MPLA (TLR4), R848 (TLR7/8), and MDP (NOD2), and an inflammatory cytokine, TNF-α (TNFR), (Figure S3A). To assess whether **2D216** was a direct agonist for the more common PRRs, we used a series of reporter cell lines expressing human receptors, including Toll-like receptors (TLRs), C-lectin-type receptors (CLRs), and the nucleotide-binding oligomerization domain-like receptors (NLRs) and cytosolic nucleic acid receptors. **2D216** did not activate any of the receptors examined (Figure S3B–E).

Although **2D216** did not function as a PRR agonist, the compound may have been intersecting with the TLR pathway through a regulatory kinase or phosphatase interaction. Screening assays for the binding of compound **2D216** to known kinases and phosphatases were conducted by commercial services using high-throughput binding assays, KINOMEscan (DiscoveRX, Eurofin Scientific) and PhosphataseProfiler (Eurofins Discovery, Eurofin Scientific) with 97 kinases (Table S2) and 22 phosphatases (Table S3), respectively. The thresholds for 35 and 50% of control kinase and phosphatase activity were considered to be active inhibition for the panels, respectively, and these values were not attained by **2D216**, indicating that these proteins did not have a significant functional interaction with the compound.

### **2D216** Activates the Ca^2^/NFAT Pathway

**Figure 5:**
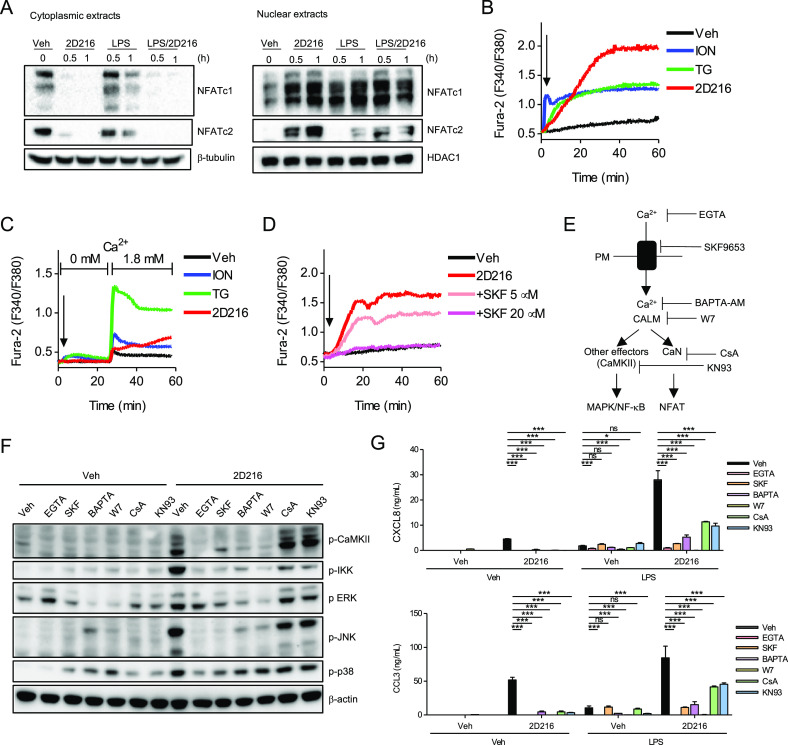
Ca^2+^/NFAT activation by **2D216**. (A) Nuclear translocation of NFATc1 and NFATc2 induced by **2D216**. THP-1 cells were incubated with **2D216** (5 μM) and/or LPS (10 ng/mL) for 0.5 and 1 h. Cytoplasmic and nuclear extracts were prepared and NFATc1 and NFATc2 were detected by immunoblot. β-tubulin and HDAC1 were used as loading controls of cytoplasmic and nuclear extracts, respectively. (B) Ca^2+^ mobilization by **2D216**. THP-1 cells were loaded with the ratiometric Ca^2+^ indicator, Fura-2, and at the time indicated by the arrow were treated with **2D216** (5 μM). ION (0.1 μM) and TG (1 μM) were used as positive controls. Time–response results of intracellular Ca^2+^ levels were recorded with a plate reader over 60 min. Data represent similar results from three independent experiments. (C) Ca^2+^ add-back assay. Fura-2-loaded THP-1 cells were treated with **2D216** (5 μM), ION (0.1 μM), and TG (1 μM) for 30 min in the absence of extracellular Ca^2+^, and then Ca^2+^ (final 1.8 mM) was added at *t* = 25 min. Data shown are representative of three independent experiments. (D) Ca^2+^ channel blocker SKF96365 reduces **2D216**-induced Ca^2+^ flux. THP-1 cells were loaded with Fura-2 and pre-treated with a broad Ca^2+^ channel inhibitor SKF96365 (SKF) for 30 min and then treated with **2D216** (5 μM) for 60 min. Data represent similar results from three independent experiments. (E) Scheme of mode of action of various Ca^2+^ signaling inhibitors. PM: plasma membrane, CALM: calmodulin, CaMKII: Ca^2+^/CALM-dependent kinase II, and CaN: calcineurin. (F) IKK and MAPK activation by **2D216** via Ca^2+^. THP-1 cells were treated with **2D216** (5 μM) for 30 min in the absence (Veh) or the presence of SKF (20 μM), extracellular Ca^2+^ chelator EGTA (2 mM), intracellular Ca^2+^ chelator BAPTA (BAPTA-AM, 50 μM), CALM inhibitor W7 (100 μM), CaN inhibitor (CsA, 40 nM), and CaMKII inhibitor (KN93, 10 μM), and immunoblot with anti-phospho antibodies against each protein was performed. (G) Cytokine secretion with Ca^2+^ inhibitors. THP-1 cells were pre-treated for 1 h with Ca^2+^ inhibitors, broad Ca^2+^ channel blocker SKF (SKF96365, 20 μM), extracellular Ca^2+^ chelator EGTA (EGTA, 2 mM), intracellular Ca^2+^ chelator BAPTA (BAPTA-AM, 50 μM), CALM inhibitor W7 (W7, 100 μM), CaN inhibitor (CsA, 40 nM), and CaMKII inhibitor (KN93, 10 μM) and treated with **2D216** (5 μM) overnight. CXCL8 and CCL3 in supernatants were determined by ELISA. Data represent mean ± SD of triplicates of two independent experiments showing similar results. *p < 0.05, **p < 0.01, ***p < 0.001, ns (not significant) by one-way ANOVA with Tukey’s *post hoc* test.

As indicated above, **2D216** induced reporter activation in the CellSensor line NFAT-bla RA1 (Table S1). Hence, activation of NFAT by **2D216** was further examined by immunoblots of cytoplasmic and nuclear extracts prepared from THP-1 cells. **2D216** induced the nuclear translocation of NFATc1 and NFATc2, the major NFAT isoforms in immune cells,^40^ as early as 30 min following stimulation (Figure 5A). In contrast, LPS treatment alone showed minimal NFATc2 nuclear translocation at 30 min (Figure 5A).

Intracellular Ca^2+^ flux plays important roles in both innate and adaptive immune responses.^41^ NFAT translocation occurs in response to changes in intracellular Ca^2+^, and our corroborating data thus directed us to examine the ability of **2D216** to induce changes in intracellular Ca^2+^. Ca^2+^ signals are orchestrated by multiple ion transport processes across the plasma membrane, endoplasmic reticulum, and inner mitochondrial membrane including active Ca^2+^ pumps, Ca^2+^/Na^+^ antiports, and diffusion. The major mechanism for Ca^2+^ entry in immune cells is via the store-operated Ca^2+^ entry (SOCE)^42^ that is activated by Ca^2+^ release from intracellular stores and involves the activation of Ca^2+^ release-activated Ca^2+^ (CRAC) channels in the plasma membrane.^43^ These CRAC channels are formed by ORAI1 proteins in the plasma membrane and activated by stromal interaction molecule (STIM)1 and STIM2 in the endoplasmic reticulum.^44^

**2D216** induced a gradual Ca^2+^ increase starting within a few minutes after the addition of **2D216** and was sustained for at least 1 h as measured by a ratiometric Ca^2+^ indicator, Fura-2, assay (Figure 5B). In the absence of extracellular Ca^2+^, **2D216** did not induce an increase in intracellular Ca^2+^ (Figure 5C), while the positive controls, ionomycin (ION) and thapsigargin (TG), induced a small increase, indicating a release of Ca^2+^ from the endoplasmic reticulum. When extracellular Ca^2+^ was replenished, ION and TG treatment resulted in a sharp increase in Ca^2+^ via the SOCE, but **2D216** did not (Figure 5B). Furthermore, BTP2, a specific inhibitor of CRAC, and siRNA knockdown of STIM1, had no effect on **2D216**-induced Ca^2+^ elevation (Figure S4A–C). The broad Ca^2+^ channel blocker, SKF96365, suppressed the **2D216**-induced Ca^2+^ elevation in a dose-dependent manner, and at 20 μM had only a partial effect on the TG-induced Ca^2+^ influx (Figure 5D and Figure S4D). These results indicated that the intracellular Ca^2+^ increase induced by **2D216** is SOCE-independent and that **2D216** induced extracellular Ca^2+^ influx via SKF96365-sensitive plasma membrane Ca^2+^ channels.

As a result of the intracellular Ca^2+^ increase, several signaling pathways and transcription factors are activated, such as the calmodulin (CALM)-calcineurin (CaN) pathway that activates NFAT, the Ca^2+^-dependent kinase-calmodulin (CaMK) pathway, and the NF-κB pathway (Figure 5E).^45^ Extracellular Ca^2+^ chelator EDTA, broad Ca^2+^ channel blocker SKF96365, intracellular Ca^2+^ chelator 1,2-Bis(2-aminophenoxy)ethane-*N*,*N*,*N*′,*N*′-tetraacetic acid tetrakis (acetoxymethyl ester; BAPTA-AM), and CALM inhibitor, W7, suppressed the **2D216**-induced phosphorylation of IKK and MAPKs (Figure 5F), suggesting that Ca^2+^ influx was also upstream of IKK/NF-κB and MAPK pathways. Of note, all inhibitors uniformly inhibited phosphorylation of CaMKII used as a control. EGTA itself enhanced the phosphorylation of ERK but suppressed that induced by **2D216**. The CaN inhibitor, cyclosporin, and the CaMKII inhibitor, KN93, minimally suppressed the phosphorylation of IKK and JNK by **2D216** compared to the other inhibitors (Figure 5G), indicating that other effectors downstream of CALM and/or CALM-independent signals may also be involved in the signaling induced by **2D216**. Functionally, SKF96365 and EGTA significantly suppressed the **2D216**-induced CXCL8 and CCL3 release in THP-1 cells (Figure 5G). Collectively, these results indicate that **2D216** increased intracellular Ca^2+^ via extracellular Ca^2+^ influx, leading to NF-κB activation via IKK/MAPK phosphorylation and NFAT nuclear translocation.

To further refine which Ca^2+^ channels might be associated with **2D216**-induced extracellular Ca^2+^ influx, we tested a panel of known Ca^2+^ channel inhibitors with **2D216** (Figure S5). Interestingly, the inhibitors for the purinergic P2X4 receptor and Na^+^/Ca^2+^ exchangers (NCX) and Na^+^/Ca^2+^/K^+^ exchangers (NCKX) both attenuated the Ca^2+^ influx induced by **2D216** (Figure S5). There was a dose-dependent inhibition of **2D216**-induced Ca^2+^ influx by two structurally unrelated P2X4R inhibitors, 5BDBD and PSB12062, and an inhibitor of NCX/NCKX, KRB7943 (Figure S6A,C). These inhibitors also abrogated **2D216**-induced cytokine production (Figure S6B,D). These two types of receptors have been reported to work in concert.^46^ The current data do not clearly support a unique target for **2D216** as there might be multiple targets that functionally induce Ca^2+^ influx.

Calcium signaling plays multiple roles in the activation, migration, and maturation of dendritic cells (DCs) that are at the intersection of the innate and adaptive immune systems.^47,48^ The calcium ionophore A23187 induced maturation and activation of DCs.^49^ Furthermore, inhibition studies using the broad Ca^2+^ channel blocker SKF96365 revealed that intracellular Ca^2+^ elevation via Ca^2+^ channels was critical for the activation of DCs by various PRR ligands such as LPS.^50^ To our knowledge, however, studies using intracellular Ca^2+^ modulators as vaccine adjuvants especially in vivo are limited. Chan et al. examined the effects of Ca^2+^ released from calcium alginate gels on DCs both in vitro and in mice and found that calcium alginate gels enhanced LPS-induced activation and maturation of DCs.^51^ Another study by An et al., using honeycomb calcium carbonate nanoparticles, demonstrated that intracellular Ca^2+^ elevation activated antigen-presenting capacity of DCs via autophagy inhibition.^52^ There are potential limitations using calcium directly or potent calcium ionophores as vaccine adjuvants including the potential for local or systemic reactogenicity given their broad action on immune cells.

### NFAT Activation by **2D216** Is Cell Type Specific

**Figure 6:**
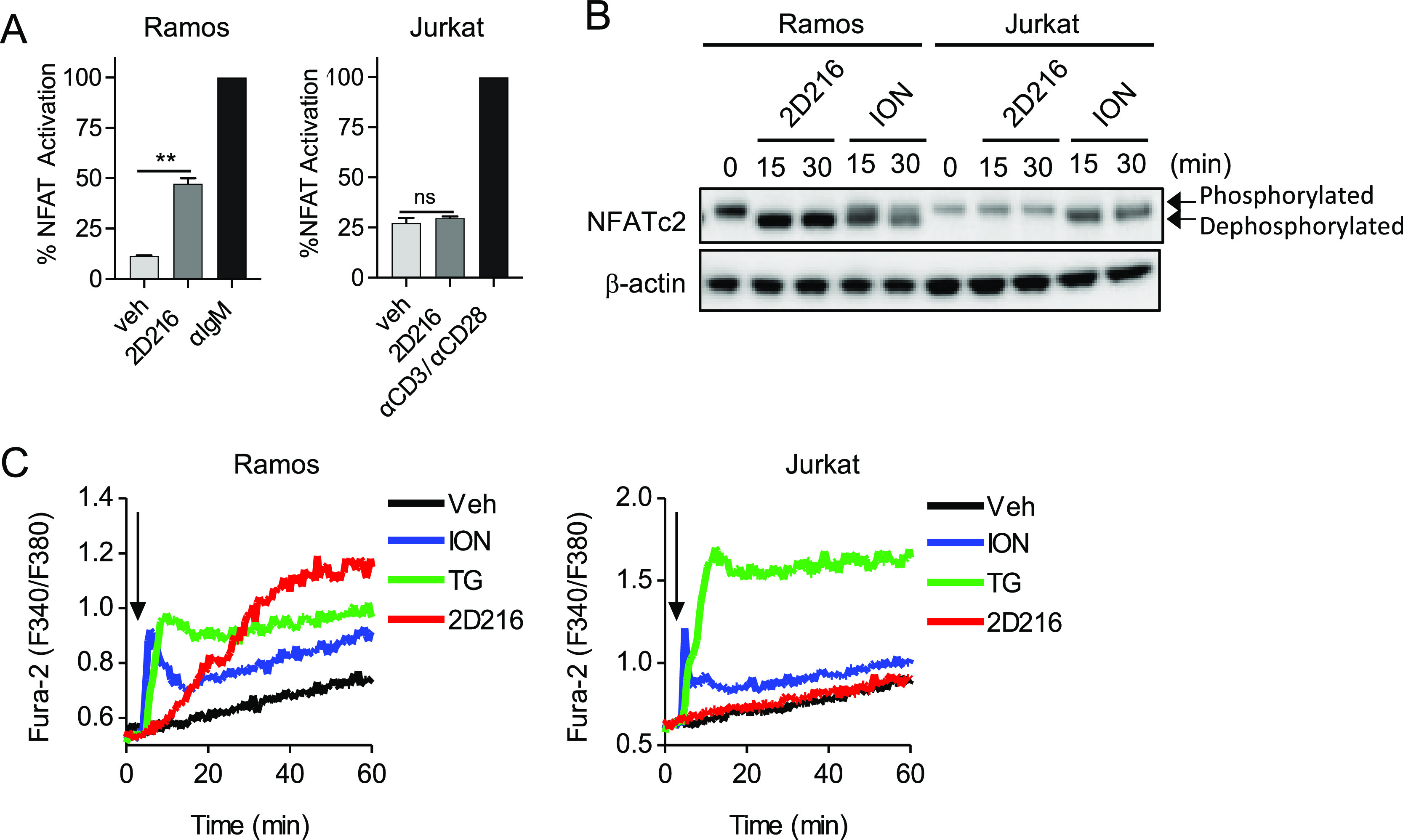
Cell-type specific Ca^2+^/NFAT activation by **2D216**. (A) The response ratios of Ramos or Jurkat NFAT reporter cells treated with 3.16 μM **2D216** were normalized to the positive controls (100%). The response ratio of anti-IgM and anti-CD3/anti-CD28 antibody-treated cells were 8.00 ± 0.41 and 3.88 ± 0.27, respectively. Data represent mean ± SD of two independent experiments showing similar results. **p < 0.01 by one-way ANOVA with Tukey’s *post hoc* test. (B) Ramos and Jurkat cells were treated with **2D216** (5 μM) or ION (1 μM) for 15 and 30 min, and NFATc2 activation was assessed by immunoblot. Slower and faster migrating bands represent phosphorylated (inactive) and dephosphorylated (active) NFATc2, respectively. (C) Cell-type specific Ca^2+^ influx by **2D216**. Ramos and Jurkat cells were loaded with Fura-2 and treated with ION (1 μM), TG (5 μM), or **2D216** (5 μM) for 60 min. The arrow indicates the addition of compounds. Data shown are representative of three independent experiments.

In the pathway analysis with **2D216** using CellSensor cell lines, we noted that the NFAT-bla reporter construct was activated in the Ramos (RA1) B cell line but not the Jurkat T cell line (Table S1). Repeated testing confirmed that **2D216** significantly activated only the Ramos-NFAT-reporter cells but not the Jurkat-NFAT reporter cells (Figure 6A). Concordantly, **2D216** induced a faster migrating form of NFATc2, corresponding to an active form dephosphorylated by CaN, in Ramos but not Jurkat cells. The positive control, ION, induced this band in both Ramos and Jurkat cells (Figure 6B). Finally, **2D216** induced gradual and sustained Ca^2+^ elevation similar to that seen in THP-1 cells in Ramos but not Jurkat cells (Figure 6C). Collectively, these results indicate that NFAT activation by **2D216** may be relatively specific to B-cells and monocytes. Interestingly, THP-1 cells that have been differentiated into a macrophage phenotype are less responsive to a **2D216** co-stimulus than the parent cells (Figure S7). Direct activation of B cells by vaccine adjuvants provides beneficial effects on host defense. For example, engagement of C3d and BAFF receptors on B cells by their ligands enhanced antibody titers against influenza virus.^53,54^ On the contrary, direct activation of T cells might lead to severe adverse effects such as cytokine storm.^55^ Hence, the activation of APCs (myeloid and B cells), but not T cells by **2D216**, is desirable for adjuvant safety.

### Selection of Analogs of **2D216**

**Figure 7:**
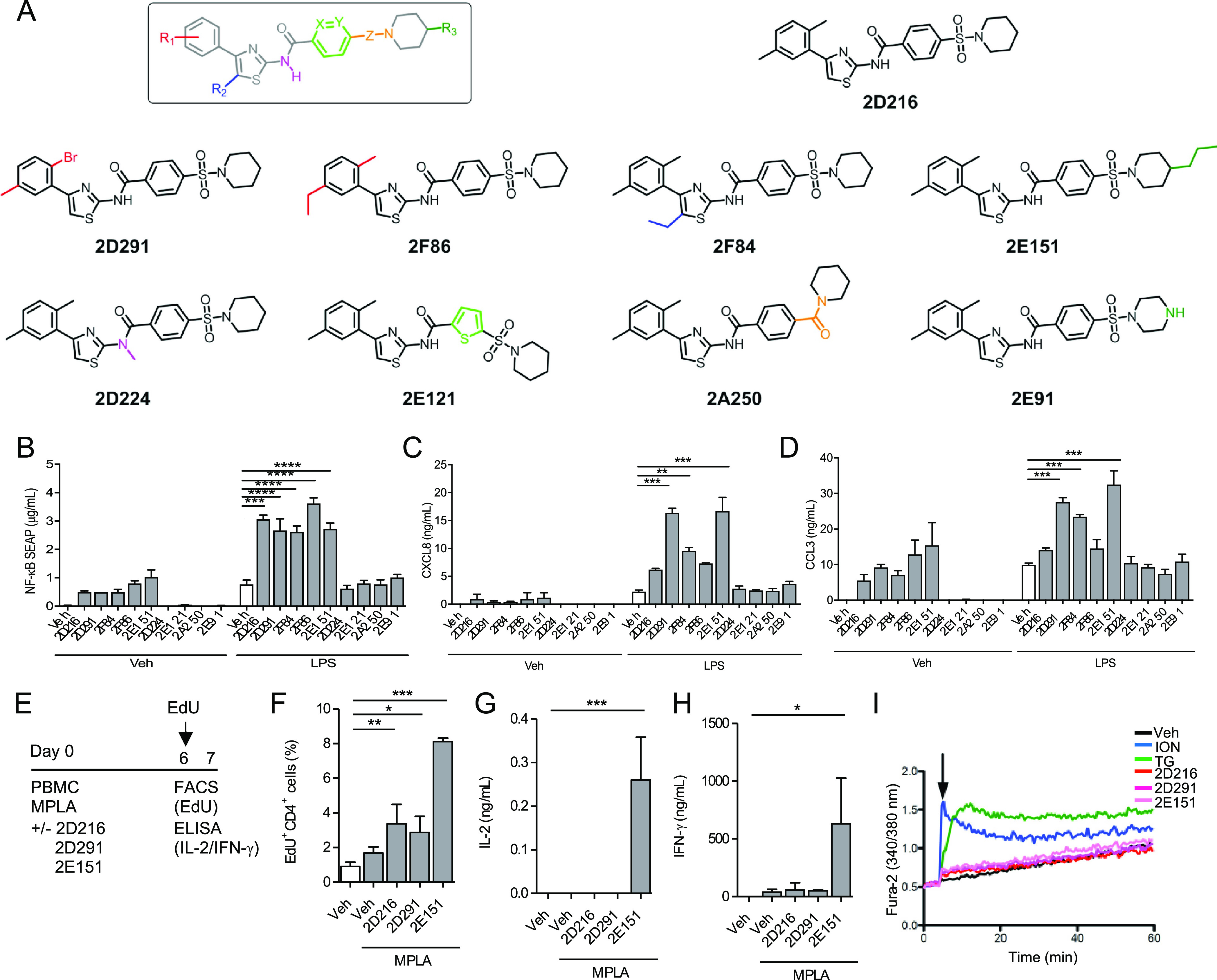
APC and T cell activation by **2D216** and analogs. (A) Chemical structures of a small exploratory series of **2D216** analogs. (B–D) NF-κB activity and cytokine secretion induced by analogs. THP1-Blue NF-κB reporter cells (0.5 × 10^6^ cells/mL) were incubated for 16 h with the vehicle (Veh), the indicated compound (5 μM), LPS (10 ng/mL), or the compound (5 μM) plus LPS (10 ng/mL). NF-κB activation was detected by quantifying the SEAP protein in the culture supernatants. THP-1 cells were treated as mentioned above, incubated for 20 h, and the levels of CXCL8 (C) and CCL3 (D) in the culture supernatants were measured by ELISA. Data presented are mean ± SD of triplicates and representative of two independent experiments showing similar results. *p < 0.05, **p < 0.01, ***p < 0.001, ns (not significant) by one-way ANOVA with Dunnett’s *post hoc* test compared to the LPS+Veh control. (E–G) **2D216** analogs enhance MPLA stimulation of autologous MLR. Human PBMC (2 × 10^6^ cells/mL) were treated with MPLA (10 ng/mL) and **2D216** or its analogs (5 μM) for 7 days. (E) EdU (10 μM) was added on day 6 to access T cell proliferation. (F) Cell suspensions were subjected to flow cytometry analysis of EdU-incorporated CD4^+^ T cells, and supernatants were assayed for (G) IL-2 and (H) IFN-γ by ELISA. Data represent mean ± SD of triplicates of two independent experiments showing similar results. *p < 0.05, **p < 0.01, ***p < 0.001, ns (not significant) by one-way ANOVA with Tukey’s *post hoc* test. (I) Ca^2+^ mobilization in Jurkat cells loaded with the ratiometric Ca^2+^ indicator, Fura-2, and at the time indicated by the arrow treated with **2D216** or analogs (5 μM). ION (0.1 μM) and TG (1 μM) were used as positive controls. The time–response pattern of intracellular Ca^2+^ levels were recorded with a plate reader over 60 min.

In a prior structure–activity relationship (SAR) study, we examined which elements of the **2D216** scaffold are necessary for enhancement of NF-κB activity.^35^ This exploratory SAR probed six different sites on the scaffold^35^ and we selected a set of representative compounds (structural differences shown in color; Figure 7A). Some of these compounds retained NF-κB activity such as **2D291**, which had a 2-bromo-5-methyl substituent on the C4-position of the thiazole ring, **2F86** bearing a 2-methyl-5-ethyl substituent on the C4-position of the thiazole ring, **2F84** with a 5-ethyl substituent on the C5-position of the thiazole ring, and **2E151**, a C4-propyl piperidine-bearing analog. These compounds were as potent as compound **2D216** and enhanced NF-κB in the presence of LPS and MPLA (Figure 7B and Figure S8A). Another set of selected compounds had modifications on the other sites of the scaffold, which led to inactivity such as **2D224** that was obtained by N-methylation of the amide nitrogen, **2E121** bearing an isosteric thiophene ring in the central core, **2A250** that replaced the sulfonamide bond with a carboxamide bond, as well as **2E91** that had a free basic amine-bearing piperazine moiety. These latter compounds did not induce NF-κB or enhance NF-κB in the presence of LPS and MPLA (Figure 7B and Figure S8A). We tested the ability of active compounds to stimulate inflammatory cytokines alone and with LPS or MPLA (Figure 7C,D and Figure S8B–D). There were different rank orders for levels of cytokine induction between the active compounds. Individual cytokines require different transcription factors (i.e., NF-κB, NFAT, etc.) for optimal production; however, among the active analogs, **2D291** (*N*-(4-(2-bromo-5-methylphenyl)thiazol-2-yl)-4-(piperidin-1-ylsulfonyl)benzamide) and **2E151** (*N*-(4-(2,5-dimethylphenyl)thiazol-2-yl)-4-((4-propylpiperidin-1-yl)sulfonyl)benzamide) were the most consistent in inducing cytokine production with LPS (Figure 7C,D) and were confirmed to augment inflammatory cytokine production in conjunction with MPLA (Figure S8B–D). Flow cytometry performed as previously described.^34^

### Enhanced T Cell Activation in a Mixed Lymphoid Reaction by **2D216** and Its Analogs

As **2D216** did not directly activate Jurkat T cells, we tested **2D216**, **2D291**, and **2E151** for their ability to enhance functional antigen presentation by murine bone marrow-derived dendritic cells (mBMDCs) (Figure S9). We used a model system with primary T cells transgenic for the T cell receptor (TCR) DO11.10 that responded to an OVA peptide on APCs. First, mBMDCs were isolated as the APCs and pretreated with a vehicle or a broad Ca^2+^ channel blocker (SKF96365) and then treated with a compound or vehicle in combination with MPLA overnight. These APCs were pulsed with OVA, washed to remove the compound and OVA, and then mixed with DO11.10 T cells. The analog-treated APCs increased T cell proliferation and IL-2 and IFN- γ production, and this activity was reduced by SKF96365 (Figure S9).

These analogs and **2D216** were also tested in a human autologous mixed lymphoid reaction (MLR) assay (a mixture of T cells and APCs) with MPLA (Figure 7E,F). **2D216**, **2D291**, and **2E151** increased the CD4^+^ T cell proliferation above that stimulated by the MPLA/vehicle control. Although **2D216** was able to augment the CD4^+^ T cell proliferation compared to vehicle control treated cells, **2E151** induced twofold greater proliferation than **2D216** (Figure 7F). **2E151** also induced significantly greater T cell cytokine release of IL-2 and IFN-γ compared to **2D216** and **2D291** (Figure 7G,H). For comparison, similar cultures of human PBMC anti-CD3/CD28 stimulation yielded % dividing cells (26 ± 0.9%, mean ± SEM), IL-2 (0.36 ± 0.06 ng/mL) and IFN-γ (323 ± 9 ng/mL). However, **2D216**, **2D291**, and **2E151** had minimal, if any, direct influence on calcium influx in Jurkat T cells (Figure 7I). These studies indicate that aminothiazoles can enhance the activation of innate immune cells by a primary stimulus like another adjuvant or TLR agonist and subsequently contribute to a greater T cell response.

## Conclusions

Using broad cell-based phenotypic HTS, we previously identified a new synthetic co-adjuvant, **2D216**, and in this study, we examined its mechanisms of action. **2D216** enhanced antigen-specific antibody responses in mice and innate immune responses induced by multiple PRR ligands. Specifically, **2D216** augmented the effect of a prototypical TLR4 ligand via a mechanism associated with extracellular Ca^2+^ influx in human cells. This co-adjuvant effect of **2D216** was cell-type specific, observed primarily in myeloid and B cells. **2D216** enhanced many desirable features for antigen presentation including upregulation of cell surface costimulatory proteins and enhanced cytokine secretion. An exploratory SAR study indicated that the analog **2E151** may have a higher potency on APCs in enhancing T cell stimulation and consequent proliferation and cytokine production. Hence, further optimization of a small molecule that modulates APC-specific Ca^2+^ influx is promising for future development of candidates as vaccine co-adjuvants.

## Materials and Methods

### Compounds

Compounds **37**, **38**, **2D216**, **50**, **171**, **172**, **173**, and **174** were purchased from Life Chemicals Inc. (Canada). **2D216** (**1**) was later resynthesized along with compounds **2D291** (**12d**), **2F86** (**12u**), **2F84** (**18r**), **2E151** (**54h**), **2D224** (**34c**), **2E121** (**42c**), **2A250** (**45c**), and **2E91** (**54i**) as reported in our structure–activity relationship (SAR) studies’ publication along with its purity and identity analysis.^35^ The numbers in the brackets for each synthesized compound refer to the compound number in the published manuscript. Purity for all the purchased compounds was verified to be more than 97% by LC–MS. The LC–MS spectra for these compounds are included in the Supporting Information. Purity analysis were done using an Agilent 1260 LC/6420 Triple Quad mass spectrometer (Santa Clara, CA) with an Onyx Monolithic C18 (Phenomenex, Torrance, CA) column.

### Cell Lines and Reagents

THP-1 (ATCC), CellSensor NF-κB-bla THP-1 (Thermo Fischer Scientific), THP1-Blue NF-κB (InvivoGen), CellSensor NFAT-bla RA-1 (Thermo Fischer Scientific), CellSensor NFAT-bla Jurkat (Thermo Fischer Scientific), and THP-1 expressing IRF3-luciferase with human RIG-I, MDA5, or STING (Thermo Fischer Scientific) were cultured in an RPMI1640 medium supplemented with 10% FBS, 100 U/mL penicillin, 100 μg/mL streptomycin, and 55 μM β-mercaptoethanol. HEK293 cells expressing the NF-κB SEAP reporter with various human TLRs (Imgenex), C-lectin receptors, or NODs (Thermo Fischer Scientific) were cultured in a DMEM medium supplemented with 10% FBS, 100 U/mL penicillin, and 100 μg/mL streptomycin. Human bloods were obtained from the San Diego Blood Bank, and PBMC were prepared using Ficoll-Paque Plus (GE Healthcare) and cultured in an RPMI1640 medium supplemented with 10% FBS, 100 U/mL penicillin, and 100 μg/mL streptomycin. Compound **2D216** was synthesized by us and its purity (>99%) was confirmed by LC/MS. LPS (LPS-EB Ultrapure), Pam3CSK4, MPLA, R848, MDP, Flagellin, CpG ODN, DAP (InvivoGen), TDP, Curdlan, and AMP were purchased from InvivoGen and used for in vitro studies. Ionomycin (Calbiochem), thapsigargin (Tocris), human recombinant TNF-α (R&D System), cyclosporin A (Calbiochem), SB203580 (Sigma-Aldrich), SP600125 (Sigma-Aldrich), LY294022 (Sigma-Aldrich), EGTA (Sigma-Aldrich), BAPTA-AM (Millipore Sigma), SKF96365 (Tocris), KN93 (Tocris), and W7 (Cayman) were used in cell culture studies. Other calcium channel inhibitors NF449, 5BDBD, PSB12062, GsMTx4, FTY720, ω-agatoxin, Capsazepine, and SNX-482 were purchased from Tocris, and A804598, A967079, KB-R7943, Nifedipine, Ruthenium red, Pyr3, and ACA were purchased from Sigma-Aldrich.

### Animals and In Vivo Immunization

Six to eight-week-old C57BL/6, BALB/c mice and D011.10 mice were purchased from The Jackson Laboratory. All animal experiments received prior approval by the UC San Diego Institutional Animal Care and Use Committee (IACUC). C57BL/6 mice (*n* = 5/group) were immunized in the right gastrocnemius muscle with OVA (20 μg/animal, Worthington Biochemical Co.) mixed with LPS (3 μg/animal, L2654, Sigma-Aldrich) and/or compound **2D216** or **50** (100 nmol/mouse) in a total volume of 50 μL on days 0 and 14. DMSO (10%) was used as the vehicle. On day 17, mice were bled and OVA-specific IgG1 and IgG2c titers were measured by ELISA as previously described.^56^

### NF-κB Reporter Cell Assays

THP-1-Blue NF-κB cells were treated for 20 h with the 5 μM compound, and the levels of NF-κB-SEAP in culture supernatants were measured by a colorimetric assay using QUANTI-Blue, a SEAP detection reagent (#rep-qbl, InvivoGen). CellSensor NF-κB-bla THP-1 cells were treated for 5 and 12 h with the 5 μM compound with or without LPS, resulting in β-lactamase production that was detected by the addition of the β-lactamase substrate LiveBLAzerTM-FRET B/G (CCF4-AM, Thermofisher Scientific) and fluorescence emission at 535 and 465 nm assayed after 3 h of incubation. Distance from LPS was calculated as the standardized percent activation values against LPS alone, calculated as the difference between percent activation values for the test compound and the mean of LPS-alone wells divided by the standard deviation for the LPS-alone wells within each plate.

### Cytokine/Chemokine ELISA and Surface Marker Flowcytometry

THP-1 cells or PBMC (1 × 10^5^ cells/well) were plated in 200 μL/well in 96-well round-bottom plates and treated with compounds overnight or compound plus LPS (10 ng/mL) or MPLA (1 μg/mL). For treatment with inhibitors, cells were pre-incubated with each inhibitor for 1 h prior to exposure to the compounds. The concentrations of compounds and inhibitors are detailed in each figure legend.

CXCL8, CCL-3, and TNF-α in the supernatant were tested by ELISA. CD40 and CD86 expression was measured using PE anti-human CD40 (BioLegends, #313006) and APC anti-human CD86 (BD bioscience #560956) by MAQSQuant Analyzer 20 (Miltenyi Biotec) and FlowJo (version 10.6.1, Becton Dickinson).

### PMA-Induced Differentiation of THP-1 Cells

THP-1 cells (1 × 10^5^ cells/well) were plated in 200 μL/well in 96-well flat-bottom plates, treated with 10 ng/mL phorbol 12-myristate 13-acetate (PMA, P1585, Sigma-Aldrich) for 48 h, and then washed. After 24 h, the PMA-differentiated THP-1 cells were treated with Veh, **2D216** alone (5 μM), LPS (10 ng/mL), and LPS plus **2D216** overnight. CXCL8 and CCL-3 in the supernatant were tested by ELISA.

### RNA-Seq and Data Analysis

THP-1 cells were treated with Veh, **2D216** alone (5 μM), LPS (10 ng/mL), and LPS plus **2D216** for 5 h, and then the total RNA was isolated. Each group has three replicates. RNA-seq was performed by the sequencing core at the La Jolla Institute for Allergy and Immunology (San Diego, CA). Briefly, single-ended sequencing was performed on the Illumina HiSeq 2500. Reads were aligned to the human reference genome (hg19) using TopHat, and mRNA expression levels were calculated per gene using HTseq. Genes were filtered if more than two-thirds of the samples had counts <10. Raw counts were then upper-quantile-normalized and used as expression values in the following analysis. Linear models for microarray (Limma, using R-limma package) were used to compare groups regarding log2 expression values. The Benjamini–Hochberg procedure was applied to control the false discovery rate (FDR). If the log2 fold change in expression for a test compound versus vehicle control was greater or less than 1 and an FDR < 0.05, the gene is considered significantly changed and used for KEGG pathway enrichment analysis (Broad Institute). Hierarchical clustering and the heat map were made using Morpheus (Broad Institute). RNA-seq data were deposited in the Array Express database at EMBLE-EBI under the accession number, E-MTAB-10222.^38^

### NanoString and Data Analysis

THP-1 cells were treated with Veh, **2D216** (5 μM), LPS (10 ng/mL), or **2D216** plus LPS for 4 h, and the total RNA was isolated. RNA was hybridized to nCounter Human Immunology Panels (NanoString, Seattle, WA) by the UC San Diego IGM Genomics Center. Data were generated in duplicates and mean of log2 transformed values were applied for further analysis.

### Immunoblot Analysis

THP-1 cells were lysed with radioimmune precipitation assay buffer (RIPA) buffer supplemented with protease inhibitor cocktail (Roche) and a phosphatase inhibitor (Millipore). For cytoplasmic and nuclear extract preparation, THP-1 cells were first lysed with hypotonic buffer (10 mM HEPES, 10 mM KCl, 0.1 mM EDTA, and 0.05% NP-40) for cytoplasmic extracts, and then nuclear extracts were prepared with nuclear extraction buffer (20 mM HEPES, 100 mM NaCl, 1 mM EDTA, and 25% glycerol). The protein concentration was determined using a Pierce BCA protein assay kit (Thermo Fischer Scientific). Ten to thirty micrograms of protein per sample was separated by SDS-PAGE, transferred to Immobilon-P PVDF membranes, and immunoblotted. Antibodies used include the following (Cell Signaling Technology): anti-phospho IKKα/β (Ser176/180) (#2697), anti-IKKβ (#8943), anti-phospho IκBα (Ser32) (#2859), anti-IκBα (#4812), anti-phospho p65 (Ser536) (#3033), anti-p65 (#8242), anti-β-actin (#3700), anti-β-tubulin (#86298), anti-phospho ERK (Thr202/Tyr204) (#4370), anti-ERK (#4695), anti-phospho p38 (Thr180/Tyr182), anti-p38 (#8690), anti-phospho JNK (Thr183/Tyr185) (#4668), anti-JNK (#9252), anti-phospho c-Jun (Ser73) (#3270), anti-c-Jun (#9165), anti-phospho CaMKII (Thr286) (#12716), and anti-Stim1 (#5668). Anti-NFATc1 (sc-7294), anti-NFATc2 (sc-7296), and anti-HDAC1 (sc-81,598) antibodies were all purchased from Santa Cruz Biotechnology.

### Calcium Influx Assay

THP-1, Ramos, and Jurkat cells were loaded with Fura-2-AM (4 μM, Abcam) in HBSS assay buffer [1× HBSS (Corning), 10 mM HEPES (pH 7.4), 1.8 mM CaCl_2_, 0.8 mM MgCl_2_, and 0.1% BSA] containing 0.04% Pluronic F127 (Thermo Fischer Scientific) at 37 °C for 40 min and at RT for additional 20 min. Fluorescence was evoked by 340 and 380 nm excitation wavelengths and emission was read at 510 nm using a fluorescence plate reader (Tecan2000). Data were presented as 340/380 fluorescence ratios representative of changes in the intracellular Ca^2+^ level.

### Murine Antigen-Specific T Cell Proliferation Assay

Murine bone-marrow-derived dendritic cells (BMDCs) (5 × 10^5^ cells/mL) from BALB/c mice were pretreated with the vehicle or broad Ca^2+^ channel blocker (SKF96365, 20 μM) and treated with vehicle **2D216** or its derivatives (5 μM) in combination with MPLA (100 ng/mL) for 24 h. BMDCs were loaded with the OVA protein (10 μg/mL) for 4 h, washed twice, and co-cultured with the same number of CFSE-labeled CD4 T cells from spleens of sex-matched DO11.10 TCR transgenic mice for 72 h. CD4^+^ T cells were isolated from spleens of DO11.10 TCR transgenic mice using an EasySep Mouse CD4 T cell isolation kit (STEMCELL Technologies, Vancouver, Canada) and labeled with CFSE (4 μM, Molecular Probe, Eugene, OR, United States). Supernatants were assayed for IFN-γ and IL-2 by ELISA, and cell suspensions were subjected to fluorescence-activated cell sorting (FACS) analysis of CFSE dilution of DO11.10 CD4 T cells. T cell division was analyzed by a MACSQuant Analyzer 10 (Miltenyi Biotec, Bergisch Gladbach, Germany) using AF647-conjugated anti-DO11.10 TCR antibodies (eBioscience, San Diego, CA, United States). The % divided, the percent of the live CFSE-labeled CD4^+^ T cells that entered division, was calculated using FlowJo (version 10.6.1, Becton Dickinson, Ashland, OR, United States).

### Mixed Lymphocyte Reaction (MLR)

Human PBMC (2 × 10^5^ cells/200 μL/well in a 96-well flat bottom plate) were treated with **2D216**, **2D291**, or **2E151** (5 μM) with or without MPLA (100 ng/mL) for 7 days. At day 6, EdU (10 μM) was added to assess CD4^+^ T cell proliferation. At day 7, culture supernatants were harvested and assayed for IL-2 and IFN-γ by ELISA. For CD4^+^ T cell proliferation, cell suspensions were incubated with FITC-conjugated anti-CD4 antibodies (eBioscience, #11–0049-42), fixed and permeabilized, and then subjected to EdU click chemistry according to the manufacturer’s protocol (Thermo Fischer Scientific). The cells were then analyzed by flow cytometry analysis using the MAQSQuant Analyzer 20 (Miltenyi Biotec) and FlowJo (version 10.6.1, Becton Dickinson).

### Direct T Cell Activation with Anti-CD3 and Anti-CD28 Antibodies

Human PBMC (2 × 10^5^ cells/200 μL/well in a 96-well flat bottom plate) labeled by CFSE (4 μM, Molecular Probe) were treated with plate-bound anti-human CD3 (1.25 μg/mL, #317302, BioLegend) and soluble anti-human CD28 (2 μg/mL, #302902, BioLegend) antibodies with or without **2D216** (5 μM) for 3 days. Supernatants were subjected to IL-2 and IFN-γ ELISA. T cell division was analyzed by the MACSQuant Analyzer 10 (Miltenyi Biotec) using APC-conjugated anti-human CD3 antibodies (#17–0038-42, eBioscience). The % divided, the percent of the live CFSE-labeled CD4^+^ T cells that entered division, was calculated using FlowJo (version 10.6.1, Becton Dickinson).

### Pathway Analysis Using CellSensor Cell Lines

Cellular pathway analysis was performed by ThermoFisher Scientific using CellSensor cell lines that were treated with graded concentrations of **2D216** from 0.315 nM to 10 μM of compound **2D216** and a positive control for each cell line as indicated in Table S1, and FRET assays were performed.

### Kinase and Phosphatase Screening

The binding of compound **2D216** to known kinases and phosphatases was conducted by commercial services using high-throughput binding assays, KINOMEscan (DiscoverX) and PhosphataseProfiler (Eurofins Discovery), respectively. The binding was calculated as % Control at 5 μM compound **2D216** = (test compound signal – positive control signal)/(negative control signal – positive control signal) × 100: negative control = DMSO (100% Control) and positive control = control compound (0% Control). Less than 35 and 50% of control kinase and phosphatase activity, respectively, were considered to be active inhibition.

### siRNA Knockdown

THP-1 cells (2 × 10^6^ cells) were transfected with 300 pmol siRNA of human STIM1 (GE Dharmacon, #D-011785-04-0010) using Nucleofector (V-001) and Nucleofector Cell Line V kits (Lonza) according to the manufacturer’s instruction. After 72 h, the knockdown efficacy was confirmed by immunoblot and the cells were used for subsequent analysis.

### Statistical Analysis

Data obtained by *in vitro* studies are shown as means with SD, and *in vivo* data are presented as means with SEM. The Mann–Whitney *U* test was used to compare two groups, and one-way ANOVA with Tukey’s *post hoc* test were used for multiple comparisons. Prism 8 software (GraphPad Software, San Diego, CA) was used. A value of *p* < 0.05 was considered statistically significant.

## Supporting Information Available

Additional pathway and compound screening data and the LC–MS spectra (PDF)
