# Supplementary material for: Small Molecule Calcium Channel Activator Potentiates Adjuvant Activity
Source: ACS Chem Biol. 2022 Jan 5;17(1):217–29. doi: 10.1021/acschembio.1c00883 (PMC8788586; doi:10.1021/acschembio.1c00883)

Supporting Information

Small molecule calcium channel activator potentiates adjuvant activity

Tetsuya Saito[1,2], Nikunj M. Shukla[1], Fumi Sato-Kaneko[1], Yukiya Sako[1], Tadashi Hosoya[1,2], Shiyin Yao[1], Fitzgerald S. Lao[1], Karen Messer[3], Minya Pu[3], Michael Chan[1], Paul J. Chu[1], Howard B. Cottam[1], Tomoko Hayashi[1], Dennis A. Carson[1], and Maripat Corr[4]*

1) Moores Cancer Center, University of California San Diego, La Jolla, California, 92093-0809, USA.
2) Department of Rheumatology, Graduate School of Medical and Dental Sciences, Tokyo Medical and Dental University (TMDU), Tokyo 113-8519, Japan
3) Herbert Wertheim School of Public Health and Longevity, University of California San Diego, La Jolla, California, 92093-0901, USA.
4) Department of Medicine, University of California San Diego, La Jolla, California, 92093-0656, USA.

*Corresponding author: Maripat Corr,

Email: mpcorr@health.ucsd.edu

Co-corresponding author: Dennis A. Carson

Email: dcarson@health.ucsd.edu

Table of Contents

Table of Contents	Page
Table S1. Pathway analysis of **2D216** using CellSensor cell lines	S3
Figure S1. Minimal enhancement of MPLA stimulation of CCL5 and CXCL10 production by **2D216**.	S3
Figure S2. Heatmap of 359 genes significantly induced by the combination of LPS and **2D216**.	S4
Table S2. Gene lists of kinases and phosphatases examined for interaction with **2D216**.	S5
Table S3. Gene lists of Phosphatases examined for interaction with **2D216**.	S6
Figure S3. Enhancement of immunostimulatory effect by pattern recognition receptors (PRR) and cytokine receptor by **2D216.**	S7
Figure S4. SOCE-independent intracellular Ca^{2+} elevation by **2D216**.	S8
Figure S5. Pharmacological characterization of Ca^{2+} channels activated by **2D216**.	S9
Figure S6. P2X4R and NCX/NCKX in **2D216** signaling	S10
Figure S7. CXCL8 and CCL3 secretion induced by **2D216** in PMA-differentiated THP-1 cells.	S11
Figure S8. Enhancement of MPLA activation of APC by **2D216** and analogs.	S11
Figure S9. Enhancement of antigen-specific T cell activation by **2D216** and its derivatives via Ca^{2+} signaling.	S12
**Materials and Methods:** LC-MS spectra for purchased compounds	S13

Table S1. Pathway analysis of 2D216 using CellSensor cell lines

Name of pathways	Name of cell line	2D216 EC50 (nM)[a)]	Positive control	EC50 (nM)
B cell receptor	NFAT-bla RA1	540 ± 5	Goat anti-Human IgM	0.523
cAMP/PKA	CRE-bla Jurkat	>10000	Forskolin	12100
DNA damage/p53 response	p53 RE-bla HCT-116	>10000	Mitomycin C	1400
ER stress	ESRE-bla HeLa	>10000	Tunicamycin	185
Glucocorticoid receptor	MMTV-bla HeLa	>10000	Dexamethasone	5.83
Hypoxia	HRE-bla ME-180	>10000	$CoCl_2$	11300
Interleukin 4/STAT6	STAT6-bla RA1	>10000	IL-4	0.00653
JAK/STAT	ISRE-bla Jurkat	>10000	IFN-α	0.000754
JAK2/STAT5	irf1-bla TF1	>10000	EPO	0.0191
MAPK	c-fos-bla ME-180	>10000	EGF	0.0596
MAPK/EGFR/Ras/Raf	AP1-bla ME-180	>10000	EGF	0.00262
Oxidative stress	ARE-bla HepG2	>10000	tBHQ	4630
PI3K/AKT/FOXO3	FOXO3 DBE-bla HeLa	>10000	Insulin	2.08
PKC/Ca^{2+}	NFAT-bla Jurkat	>10000	Thapsigargin	7.01
T cell receptor	NFAT-bla Jurkat	>10000	anti-CD3/anti-CD28	0.105
TNF-α/JNK	AP1-bla ME180	>10000	TNF-α	0.00393

[a)] Cells were treated with increasing concentrations of 2D216, and positive controls and FRET assay was performed to determine EC50.

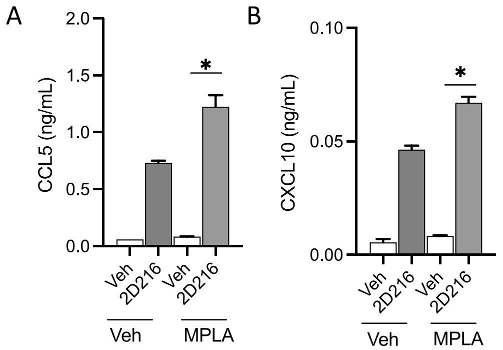

Figure S1. Minimal enhancement of MPLA stimulation of CCL5 and CXCL10 production by 2D216. THP1 cells (0.5×10^6 cells/mL) were incubated for 20 h with vehicle (Veh), **2D216** (5 μM), MPLA (1 μg/mL) or **2D216** (5 μM) plus MPLA (1 μg/mL) and the levels of CCL5(A) and CXCL10 (B) in the culture supernatants were measure by ELISA. Data presented are mean ± SD of triplicates and representative of two independent experiments showing similar results. *p≤0.05, significant by Mann-Whitney *U* test.

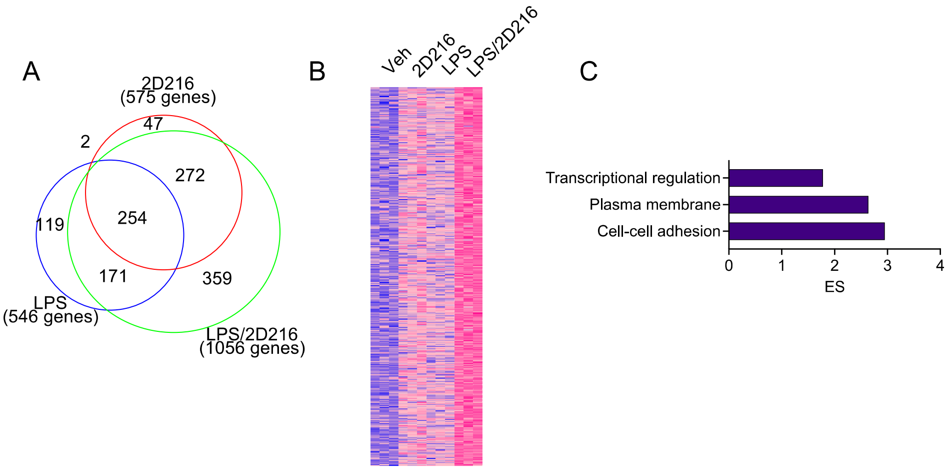

Figure S2. RNA-seq analysis of genes and gene sets enriched by 2D216 in combination with LPS. (A) Venn diagram of up-regulated genes (log2 FC>1 and FDR<0.05) by stimulation with **2D216** (5 μM) and/or LPS (10 ng/mL) for 5 h in THP-1 cells. (B) Heatmap of 359 genes up-regulated by **2D216** in combination with LPS. (C) Gene-enrichment and DAVID functional annotation analysis of up-regulated genes by the combination of **2D216** and LPS. Three representative gene sets (FDR<0.1), one from each of the three functional annotation clusters with DAVID enrichment scores (ES)≥1.8, are shown. Upregulated genes that are included in the identified pathways include:

SPEN, NANOS1,KLF10, TSHZ3, PCGF5, NFATC1, AHR, WTIP, RUNX1, PURB, MNT, ATXN1, ELF3, MDFIC, MIER2, BEND3, ZNF503, SNAI2, MSX1, ZBTB7A, ZFPM1, BEND5, HES4, NKX3-1; CNN3, FM129B, SMAD7, TJP1, PPFIBP1, MICALL1, TNKS1BP1, TBC1D10A, PLEC; KCNG1, CLIC6, GPSM1, NRP1, DOCK4, DAGLA, CD82, TFRC, RASGRF2, MAST2, SLC2A1, ECE1, SLC7A11, JPH1, FGFRL1, CXCL16, IL27RA, PLAU, NCS1, FLVCR1, SLC16A9, PLAUR, ENPP5, ARRDC4, VAV3, PAQR9, ANXA2, ARRDC3, NIPA1, LIG4, OSMR, ATP1B1, ADRA2B, RAP2C, ADAM17, MRAS, TRAF4, PRKAR1B, ITGA6, ADAM8, SHANK3, DSC2, EPHA2, NOTCH3, SEMA7A, TTYH3, PCDH10, ABHD6, ATP10A, TNFRSF11A, FAM129B, DPP4, HRH1, GPR153, FLRT2, GNG4, PIP5K1A, CD59, FLNC, S1PR2, LY6G6C, ATP9A, JAM2, GPR157, ABCA1, SVIL, P2RY11, AOC2, MYO10, CAV2, CAV1, KCNJ14, PCDHGC3, IL31RA, TNFRSF10A, SYNJ2, GNG12, BAIAP2, ACVR2A, TJP1, CCDC88A, PTPRE, MYO1C, MDM2, SPIRE1, SIGLEC1, TJP2.

Table S2. Gene lists of kinases and phosphatases examined for interaction with **2D216.**

Gene Symbol	%Control at 5 μM[a)]	Gene Symbol	%Control at 5 μM[a)]
ABL1(E255K)-phosphorylated	79	KIT(V559D,T670I)	100
ABL1(T315I)-phosphorylated	88	LKB1	82
ABL1-nonphosphorylated	75	MAP3K4	87
ABL1-phosphorylated	97	MAPKAPK2	96
ACVR1B	97	MARK3	100
ADCK3	67	MEK1	100
AKT1	100	MEK2	100
AKT2	100	MET	100
ALK	100	MKNK1	100
AURKA	100	MKNK2	100
AURKB	78	MLK1	100
AXL	100	p38-alpha	100
BMPR2	96	p38-beta	92
BRAF	100	PAK1	100
BRAF(V600E)	100	PAK2	100
BTK	100	PAK4	100
CDK11	86	PCTK1	92
CDK2	100	PDGFRA	100
CDK3	100	PDGFRB	100
CDK7	100	PDPK1	100
CDK9	100	PIK3C2B	100
CHEK1	100	PIK3CA	100
CSF1R	100	PIK3CG	96
CSNK1D	100	PIM1	100
CSNK1G2	100	PIM2	100
DCAMKL1	100	PIM3	100
DYRK1B	71	PKAC-alpha	100
EGFR	100	PLK1	73
EGFR(L858R)	100	PLK3	100
EPHA2	85	PLK4	97
ERBB2	100	PRKCE	100
ERBB4	97	RAF1	100
ERK1	100	RET	100
FAK	85	RIOK2	100
FGFR2	98	ROCK2	100
FGFR3	93	RSK2(Kin.Dom.1-N-terminal)	51
FLT3	99	SNARK	100
GSK3B	91	SRC	100
IGF1R	100	SRPK3	98
IKK-alpha	88	TGFBR1	100
IKK-beta	84	TIE2	100
INSR	100	TRKA	100
JAK2(JH1domain-catalytic)	77	TSSK1B	89
JAK3(JH1domain-catalytic)	100	TYK2(JH1domain-catalytic)	51
JNK1	75	ULK2	95
JNK2	96	VEGFR2	100
JNK3	100	YANK3	93
KIT	100	ZAP70	90
KIT(D816V)	100		

[a)] Threshold for kinase inhibition was <35% percent control

Table S3. Gene lists of Phosphatases examined for interaction with **2D216.**

Gene Symbol	% Control at 5 μM[a)]
CD45	86
DUSP22	82
HePTP	89
Lambda PP	87
LMPTP-A	101
LMPTP-B	97
MKP5	96
PP1α	105
PP2A	90
PP5	78
PTP MEG1	87
PTP MEG2	94
PTP-1B	84
PTPN22	91
PTPβ	96
RPTPμ	104
SHP-1	97
SHP-2	103
TCPTP	100
TMDP	102
VHR	87
YopH	95

[a)] Threshold for kinase inhibition was <50% percent control

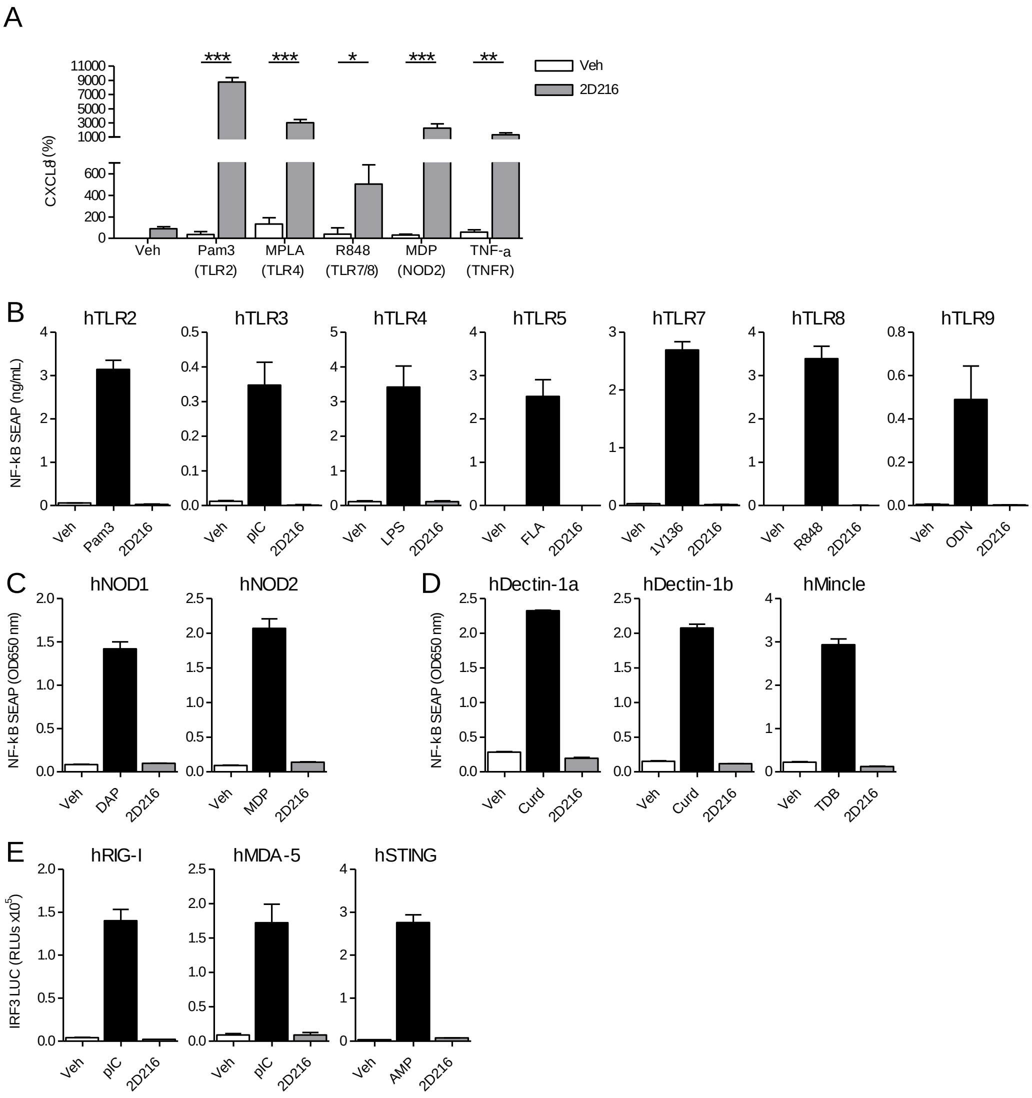

Figure S3. Enhancement of immunostimulatory effect by pattern recognition receptors (PRR) and cytokine receptor by 2D216. (A) THP-1 cells were treated with Veh, **2D216** (5 µM) and/or PRR ligands including Pam3CSK4 (Pam3) for TLR2 (10 ng/mL), MPLA for TLR4 (100 ng/mL), R848 for TLR7/8 (10 µg/mL), the muramyl dipeptide (MDP) for NOD2 (10 µg/mL), and TNF-α for TNF receptor (TNFR) (2 ng/mL) for 20 h and the culture supernatants were subjected to CXCL8 ELISA. The mean value obtained with Veh plus **2D216** was defined as 100% (CXCL8 = 0.13 ± 0.02 ng/mL). (B-E) **2D216** is not a PRR agonist. (B-D) HEK293 cells expressing the NF-κB SEAP reporter with human TLRs (B), with human NODs (C), or with human CLRs (D) were treated with **2D216** (5 µM) or their ligand as a positive control and NF-κB activation was detected by SEAP in the culture supernatants. (E) HEK293 cells expressing the IRF3 luciferase reporter with human RIG-I or MDA5, or THP-1 cells expressing the IRF3 luciferase reporter with human STING were treated with **2D216** (5 µM) or their ligand as a positive control and IRF3 activation was detected by luciferase assay. Data represents mean ± SD of triplicates and is representative of two independent experiments showing similar results. *p<0.05, **p<0.01, ***p<0.001 by one-way ANOVA with Tukey's *post hoc* test.

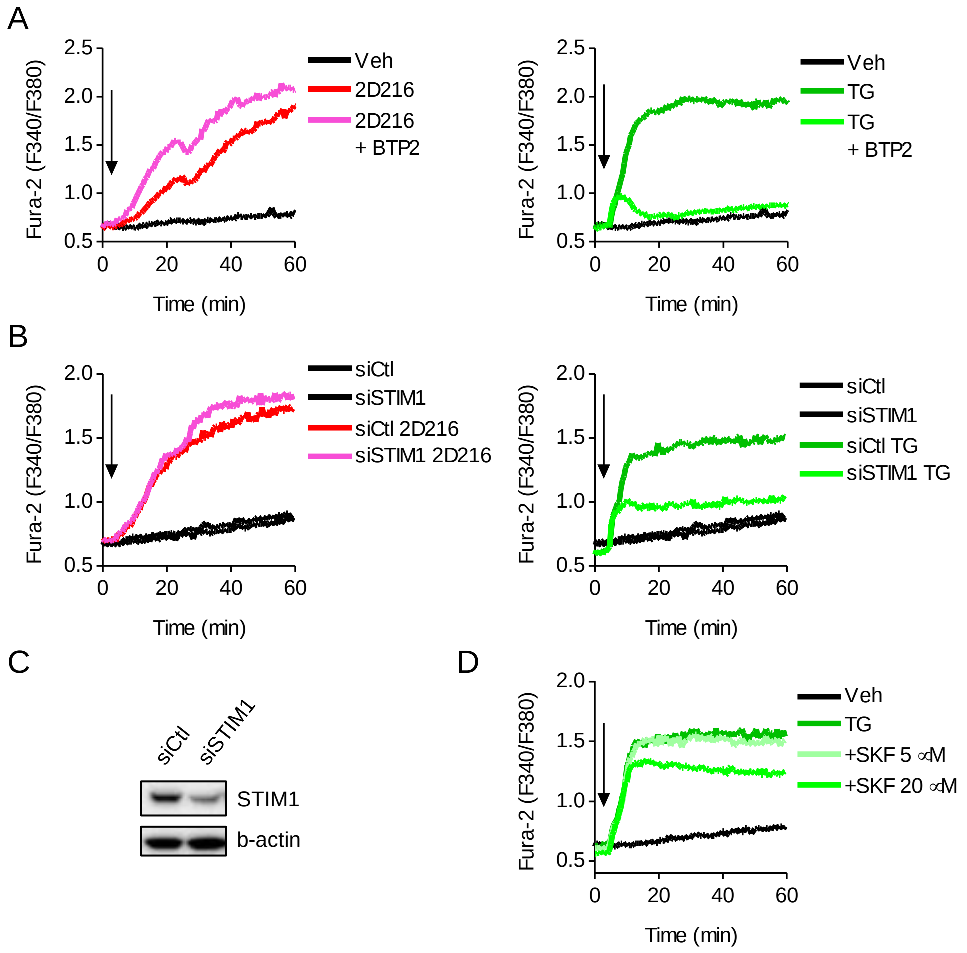

Figure S4. SOCE-independent intracellular Ca^{2+} elevation by 2D216. (A) Fura-2-loaded THP-1 cells were pre-treated with SOCE inhibitor, BTP2 (1 µM) for 30 min and treated with **2D216** (5 µM) or thapsigargin (TG, 1 µM) for 60 min. Data represent typical results from three independent experiments. (B) Effects of STIM1 knockdown. Fura-2-loaded THP-1 cells transfected with siCtl or siSTIM1 were treated with **2D216** (5 µM) or thapsigargin (TG, 1 µM) for 60 min. Data represent typical results from three independent experiments. (C) Knockdown of STIM1, a component of SOCE, by siRNA. THP-1 cells were transfected with STIM1-targeted (siSTIM1) or control (siCtl) siRNAs and the expression of STIM1 was assessed by immunoblot. (D) Effects of broad Ca^{2+} channel blocker SKF96365 on thapsigargin (TG)-induced Ca^{2+} influx. Fura-2-loaded THP-1 cells were pre-treated with SKF96365 (SKF, 5 and 20 µM) for 30 min and treated with TG (1 µM) for 60 min. Data represent typical results from three independent experiments.

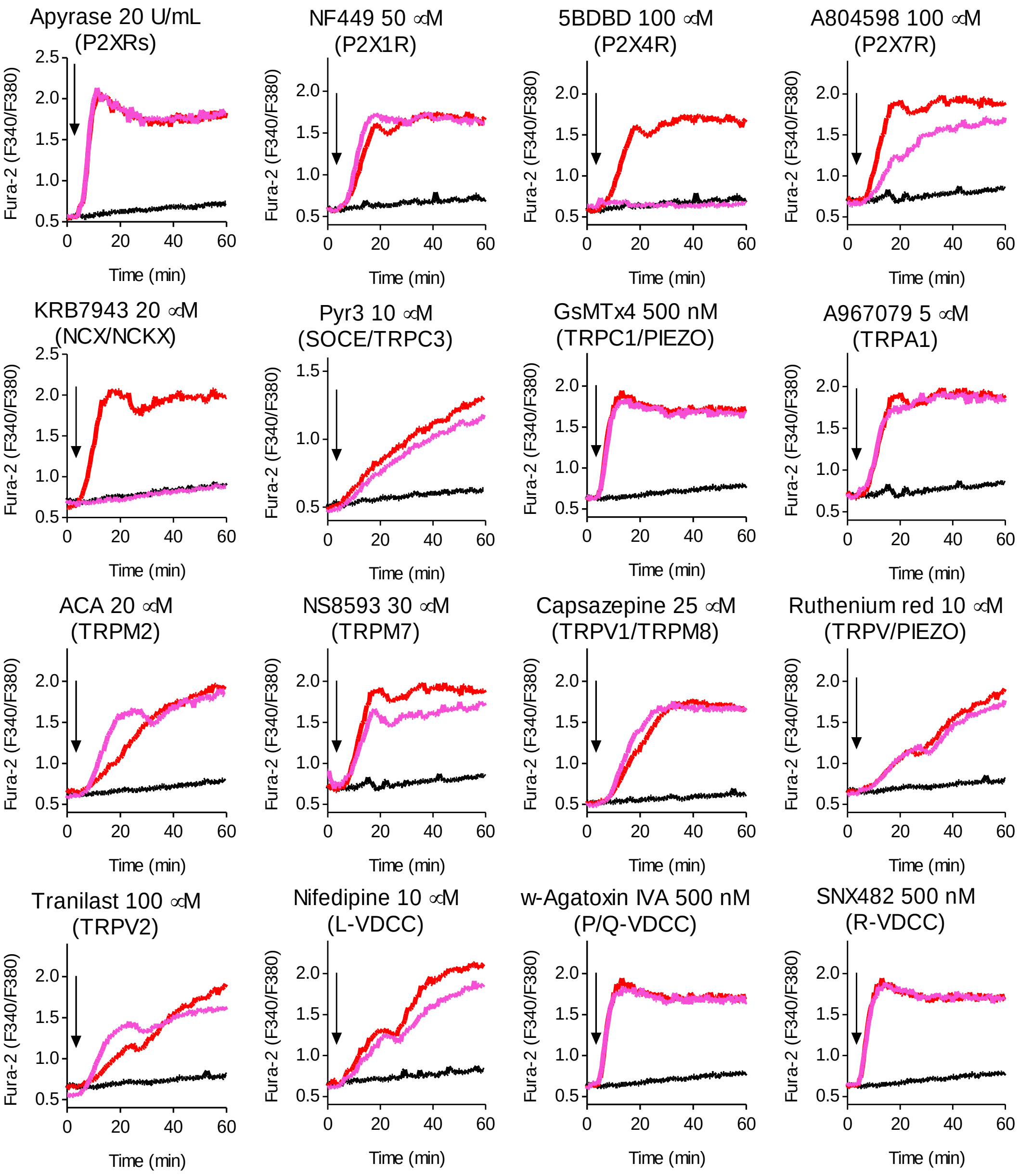

Figure S5. Pharmacological characterization of Ca^{2+} channels activated by **2D216**. Fura-2-loaded THP-1 cells were pre-treated with the indicated inhibitors and treated with **2D216** (5 µM) for 60 min: Apyrase (P2R inhibitor), NF449 (P2X1R inhibitor), 5BDBD (P2X4R inhibitor), and A804598 (P2X7R inhibitor), KRB7943 (NCX/NCKX), Pyr3 (SOCE/TRPC3 inhibitor), GsMTx4 (TRPC1 inhibitor), A967079 (TRPA1 inhibitor), ACA (TRPM2 inhibitor), NS8593 (TRPM7 inhibitor), Capsazepine (TRPM8/TRPV1 inhibitor), Rutenium red (TRPV inhibitor), Tranilast (TRPV2 inhibitor), Nifedipine (L-VDCC inhibitor), Omega-Agatoxine IVA (P/Q-VDCC inhibitor), SNX482 (R-VDCC inhibitor). Black line: vehicle control, red line: **2D216** alone, pink line: **2D216** with the indicated inhibitor. Data shown are representative of three independent experiments.

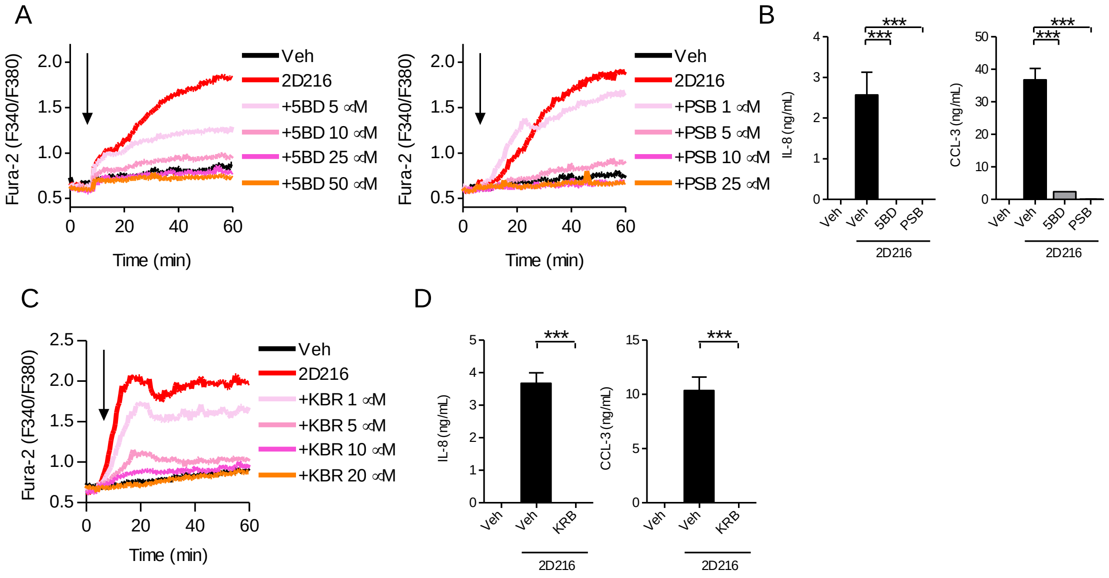

Figure S6. P2X4R and NCX/NCKX in 2D216 signaling. (A) Dose dependent inhibition of **2D216**-induced Ca^{2+} influx by two structurally unrelated P2X4R inhibitors, 5BDBD (5BD) and PSB12062 (PSB). Fura-2-loaded THP-1 cells were pre-treated with increasing concentrations of 5BDBD or PSB12062 for 30 min and treated with **2D216** (5 µM) for 60 min. Arrow indicates the time of stimulation. Data represent typical results from three independent experiments. (B) Cytokine secretion with P2X4R inhibitors. THP-1 cells were pre-treated for 1 h with 5BDBD (20 µM) or PSB12062 (10 µM) and treated with **2D216** (5 µM) overnight. IL-8 (CXCL8) and CCL3 in supernatants were determined by ELISA. (C) Dose dependent inhibition of **2D216**-induced Ca^{2+} influx by KRB7943 (KRB) inhibitor of NCX and NCKX exchangers. (D) Inhibition of THP-1 cytokine production induced by **2D216** (5 µM) with KRB7943 (10 µM) overnight. IL-8 (CXCL8) and CCL3 in supernatants were determined by ELISA. Data represent mean ± SD of triplicates of two independent experiments showing similar results. ***P<0.001by one-way ANOVA with Tukey’s post hoc test.

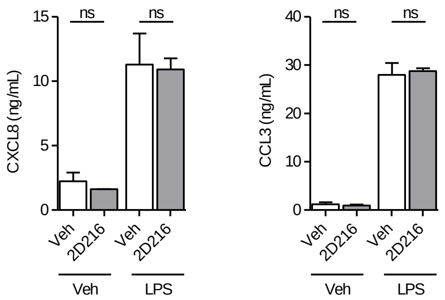

Figure S7. CXCL8 and CCL3 secretion induced with 2D216 in PMA-differentiated THP-1 cells. PMA-differentiated THP-1 cells were incubated with Veh, **2D216** (5 µM), LPS (10 ng/mL), or **2D216** (5 µM) plus LPS (10 ng/mL) for 20 h and the levels of CXCL8 and CCL3 in the culture supernatants were measured by ELISA. Data presented are mean ± SD of triplicates and are representative of two independent experiments showing similar results. ns-not significant by Mann-Whitney *U* test.

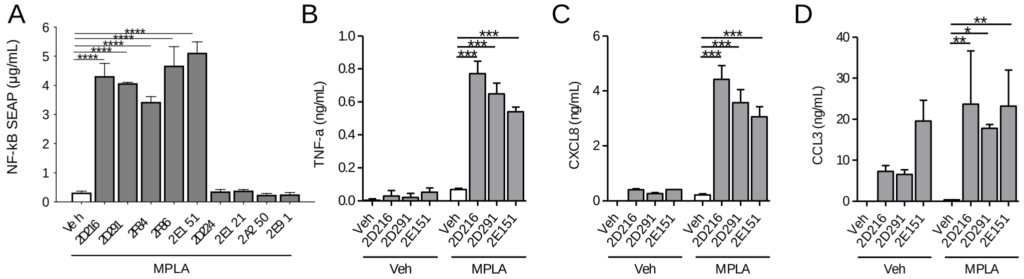

Figure S8. Enhancement of MPLA activation of APC by 2D216 and analogs. NF-κB activity and cytokine secretion induced by analogs. (A) THP1-Blue NF-κB reporter cells (0.5×10^6 cells/mL) were incubated for 20 h with vehicle (Veh), the indicated compound (5 µM), MPLA (1 µg/mL) or compound (5 µM) plus MPLA (1 µg/mL). NF-κB activation was detected by quantifying SEAP protein in the culture supernatants. THP-1 cells were treated as above, incubated for 20 h and the levels of TNF-α (B), CXCL8 (C) and CCL3 (D) in the culture supernatants were measured by ELISA. Data presented are mean ± SD of triplicates and representative of two independent experiments showing similar results. *p<0.05, **p<0.01, ***p<0.001, ns (not significant) by one-way ANOVA with Dunnett's *post hoc* test compared to the MPLA+ Veh control.

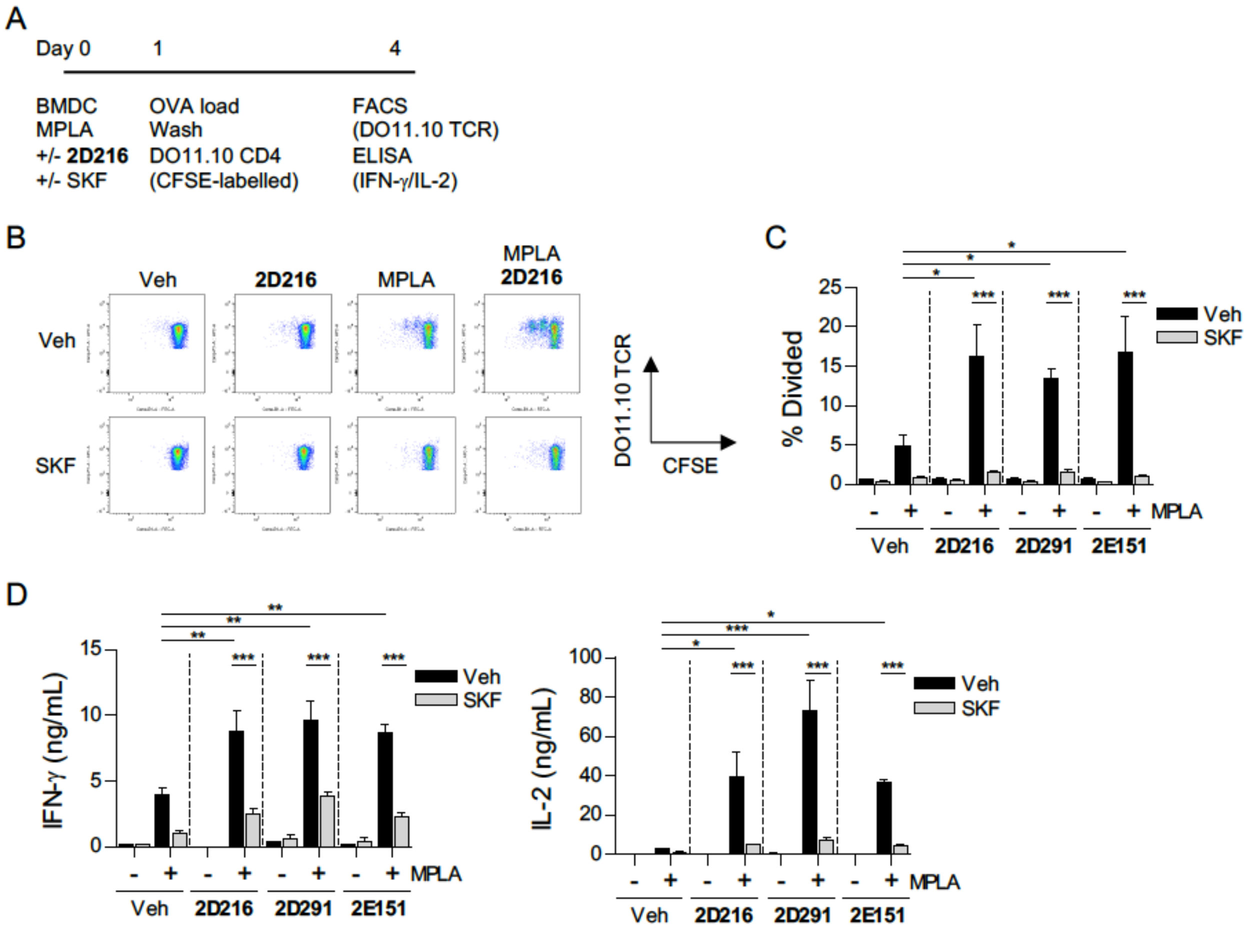

Figure S9. Enhancement of antigen-specific T cell activation by 2D216 and its derivatives via Ca^{2+} signaling. (A) Experimental scheme where mouse bone marrow-derived dendritic cells (BMDCs) were pretreated with Veh or broad Ca^{2+} channel blocker (SKF96365, SKF, 20 μM) and treated with Veh, **2D216**, its derivatives (5 μM) in combination with MPLA (100 ng/mL) for 24 hr. BMDCs were loaded with OVA protein (10 μg/mL) for 4 h, washed twice, and co-cultured with the same number of CFSE-labelled CD4 T cells from spleens of sex-matched DO11.10 TCR transgenic mice for 72 h. Supernatants were subjected to IFN-γ and IL-2 ELISA and cell suspensions were subjected to FACS analysis of CFSE dilution of DO11.10 CD4 T cells. (B and C) Representative dot plots (B) of proliferated CFSE-low DO11.10 CD4 T cells treated as indicated and quantification of % divided (CFSE-diluted) cells (C). Data represent mean ± SD of triplicates of two independent experiments showing similar results. *P<0.05, **P<0.01, ***P<0.001, ns (not significant) by one-way ANOVA with Tukey's post hoc test. (D) IFN- γ and IL-2 in supernatants were determined by ELISA. Data represent mean ± SD of triplicates of two independent experiments showing similar results. *P<0.05, **P<0.01, ***P<0.001 by one-way ANOVA with Tukey's post hoc test.

Materials and Methods

LC-MS spectra of purchased compounds

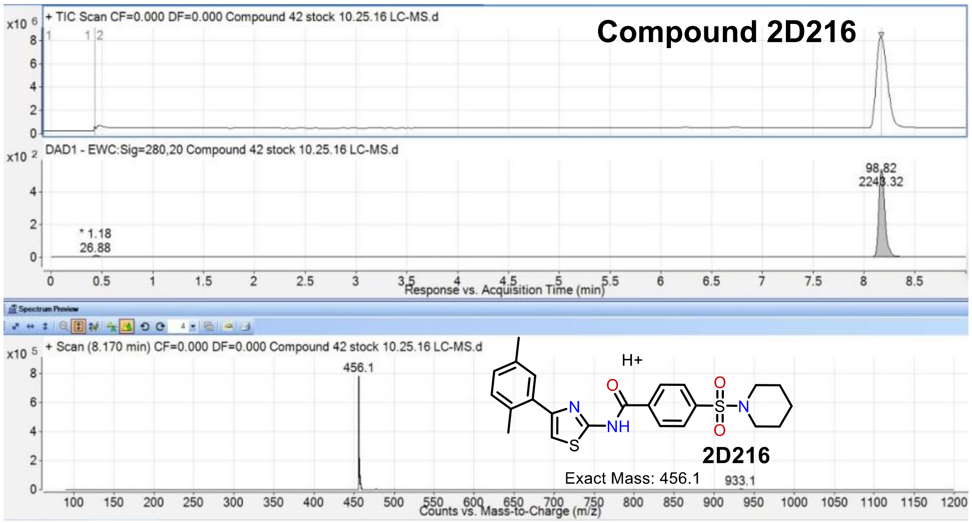

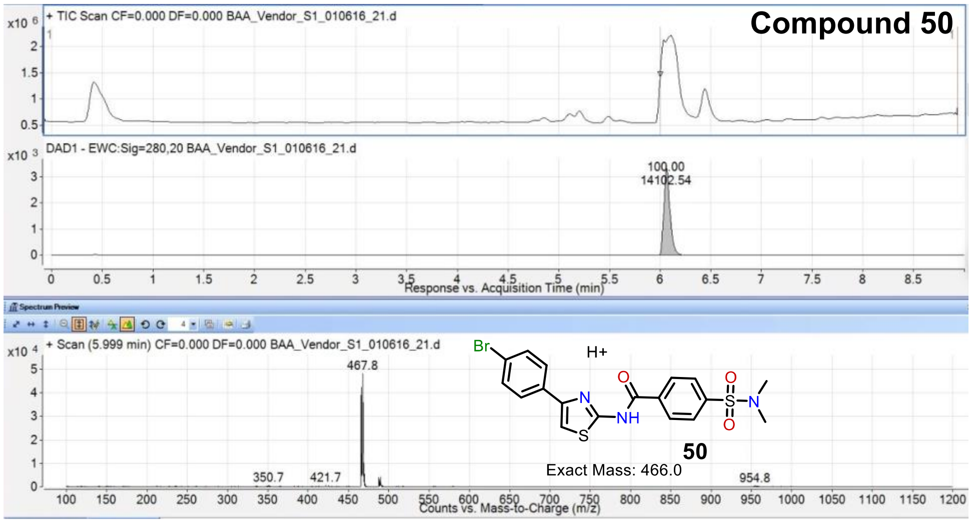

Supplement: Supplementary file 1 — cb1c00883_si_001.pdf [file cb1c00883_si_001.pdf]
